# The stability of Fbw7α in M-phase requires its phosphorylation by PKC

**DOI:** 10.1371/journal.pone.0183500

**Published:** 2017-08-29

**Authors:** Sihem Zitouni, Francisca Méchali, Catherine Papin, Armelle Choquet, Daniel Roche, Véronique Baldin, Olivier Coux, Catherine Bonne-Andrea

**Affiliations:** 1 Centre de Recherche de Biologie Cellulaire de Montpellier, CNRS, UMR 5237, Université de Montpellier, Montpellier, France; 2 Institut de Génétique Humaine, CNRS, UMR 9002, Université de Montpellier, Montpellier, France; 3 Institut de Génomique Fonctionnelle, CNRS UMR 5203, Université de Montpellier, Montpellier, France; 4 Institut de Biologie Computationnelle, LIRMM, CNRS, Université de Montpellier, Montpellier, France; Institut de Genetique et Developpement de Rennes, FRANCE

## Abstract

Fbw7 is a tumor suppressor often deleted or mutated in human cancers. It serves as the substrate-recruiting subunit of a SCF ubiquitin ligase that targets numerous critical proteins for degradation, including oncoproteins and master transcription factors. Cyclin E was the first identified substrate of the SCF^Fbw7^ ubiquitin ligase. In human cancers bearing *FBXW7*-gene mutations, deregulation of cyclin E turnover leads to its aberrant expression in mitosis. We investigated Fbw7 regulation in *Xenopus* eggs, which, although arrested in a mitotic-like phase, naturally express high levels of cyclin E. Here, we report that Fbw7α, the only Fbw7 isoform detected in eggs, is phosphorylated by PKC (protein kinase C) at a key residue (S18) in a manner coincident with Fbw7α inactivation. We show that this PKC-dependent phosphorylation and inactivation of Fbw7α also occurs in mitosis during human somatic cell cycles, and importantly is critical for Fbw7α stabilization itself upon nuclear envelope breakdown. Finally, we provide evidence that S18 phosphorylation, which lies within the intrinsically disordered N-terminal region specific to the α-isoform reduces the capacity of Fbw7α to dimerize and to bind cyclin E. Together, these findings implicate PKC in an evolutionarily-conserved pathway that aims to protect Fbw7α from degradation by keeping it transiently in a resting, inactive state.

## Introduction

Cells rely on the ubiquitin-proteasome system to mediate the regulated degradation of protein and maintain cellular homeostasis. In this process, one key family of ubiquitin ligases are the SCF (Skp1/Cul-1/F-box) complexes, in which F-box-bearing proteins act as substrate-recruiting factors [1]. Fbw7 (also known as Fbxw7, hCdc4, hAgo or Sel-10) is an F-box protein that controls the stability and thus the levels of numerous proteins including potent oncoproteins [2, 3]. With the exception of cyclin E [4, 5], Mcl1 [6, 7] and Aurora A [8], the substrates of Fbw7 are master transcriptional regulators including c-Myc [9, 10], c-Jun [11], JunB [12, 13], Notch proteins [14], MED13 [15], KLF5 [16, 17], KLF2 [18], mTOR [19], PCG-1α [20], C/EBPδ [21, 22], TGIF1 [23], NFKB2/p100 [24, 25], NRF3 [26], Hif1[27], and HSF1 [28]. As a consequence of its critical role, alteration of Fbw7 functions leads to defects in cellular proliferation, differentiation, apoptosis and metabolism, and to the deregulation of numerous pathways with oncogenic potential [2, 29, 30]. Functionally, Fbw7 is a haploinsufficient tumor suppressor [31], and deletions, promoter hypermethylation or mutations of the gene are found in many human cancers. Its role as a tumor suppressor was further demonstrated by genetic ablation of Fbw7 in mice (reviewed in [29, 30, 32]).

The human *FBXW7* gene on chromosome 4q32 comprises 11 exons and encodes three different isoforms Fbw7α, -β and -γ, due to the expression of three mRNAs that employ distinct 5′ exons [33]. Transcription at each of the three alternative first exons is independently regulated by specific transcription factors. For example, p53 upregulates Fbw7β, while Hes5 attenuates its expression [34–36], and the α-isoform is indirectly repressed by presenilin [37] and directly by C/EBP [38]. Fbw7 expression is also regulated by different oncogenic microRNAs such as miR-27a, miR-92a and miR-223 in numerous cancers (reviewed in [39]).

The 5′ exons encode signals that direct the isoforms to distinct subcellular compartments: Fbw7α in the nucleoplasm, Fbw7β in the cytoplasm and Fbw7γ in the nucleolus [40]. The region common to the three Fbw7 isoforms contains three important functional domains: a D-domain to mediate Fbw7 dimerization which regulates substrate interactions and ubiquitylation, an F-box domain to mediate Skp1 binding and assembly of the SCF ubiquitin ligase, and a WD40-repeat domain that binds substrates [41–44].

Fbw7 is subjected to post-translational modifications. Firstly by ubiquitylation in an autocatalytic reaction within the SCF complex that is regulated by dimerization of Fbw7 via the D-domain [44]. In addition, the autocatalytic ubiquitylation of Fbw7 can be antagonized by the deubiquitinase Usp28 [45]. Fbw7 can also be regulated through phosphorylation at serine/threonine residues shared by the three isoforms which influence differentially its stability. For example, ERK kinase phosphorylates Fbw7 at T205; this is required for its interaction with the Pin1 peptidyl-prolyl cis-trans isomerase and leads to its ubiquitylation and proteosomal degradation, and consequently to increased levels of c-Myc and Mcl1 in cancer cells [46, 47]. In the same way, Plk2-dependent phosphorylation at S176 induces destabilization of Fbw7 and the concomitant accumulation of cyclin E [48]. In contrast, PI3K- and Sgk1-dependent phosphorylation of Fbw7 at S227 results in increased levels of Fbw7 and decreased levels of cyclin E, c-Myc and Notch1 respectively [49, 50]. To date, only two phosphorylation events have been shown to be isoform-specific: a PKC-mediated phosphorylation of S10, and S18 in the unique N-terminal region of the α-isoform [51]. Fbw7 localizes to the nucleoplasm via two nuclear localization signals (NLS), one in the unique N-terminal region (NLS1) and one in the common region (NLS2) [40]. In the absence of a functional NLS2, a phosphomimetic aspartate mutation of S10, but not of S18, delocalizes Fbw7 in the cytoplasm [51].

Cyclin E associates with Cdk2 to drive cell proliferation by regulating the G1/S phase transition of the metazoan cell cycle [52]. Deregulated cyclin E expression has been linked to replicative stress and genomic instability [53–55]. Cyclin E expression is periodic: it accumulates in late G1, peaks at G1/S, and declines during S phase. Fbw7 is crucial for the maintenance of cyclin E periodicity in normal cell cycles, since its functional inactivation leads to increased cyclin E levels at all cell cycle phases, including mitosis [56]. However, accumulation of cyclin E during a mitotic-like phase does occur naturally during meiotic maturation of the *Xenopus laevis* oocyte, the final stage of oogenesis [57, 58]. In fertilizable eggs arrested in metaphase II (MII), large amounts of phosphorylated and stable cyclin E are stockpiled within active cyclin E/Cdk2 complexes. Investigation of the causes of this egg specificity led us to the discovery of a novel mechanism of regulation of Fbw7α. We identify one residue of Fbw7α (S18) that is phosphorylated by PKC in a manner coincident with its inactivation towards cyclin E in eggs arrested in metaphase II. S18 was previously shown to be targeted by multiple members of the PKC family *in vitro* and in mammalian cells [51]. However, a functional role for S18 phosphorylation has not been identified. Here we show that phosphorylation of this site occurs when the compartmentalization between Fbw7α and PKC is abrogated upon nuclear envelope breakdown in M-phase and importantly, that this phosphorylation event is important to inactivate and stabilize the F-box during this cell-cycle phase.

## Materials and methods

### *Xenopus* oocytes

*Xenopus laevis* frogs were housed at the CRBM (Centre de Recherche de Biologie Cellulaire de Montpellier), which has an institutional agreement (number A34-172-39) from the Direction Départementale de la Protection des Populations (DDPP) de l’Hérault (France), operating under the supervision of the Ministry of Agriculture. The protocol was not further submitted to the approval of an ethics committee, as such approval was not necessary for these experiments under French and European legislation at the time they were conducted. Mature oocytes were collected from females that had been injected with 0.5 ml (500 IU) of human chorionic gonadotropin (hCG) into the dorsal lymph sac. Females began laying eggs 12 hr after the hCG injection and were used again after a recovery period of six months. The procedure to remove oocytes of stages VI was conducted under Tricaine anesthesia (MS222, 2 g/l). The ovarian lobes were removed through a small abdominal incision. Surgical oocyte harvest was performed once per year on each frog for up to three years. After this period, frogs were euthanized by immersion in a buffered solution of Tricaine (MS222, 4 g/l). The follicular cell layer of stage VI oocytes isolated from freshly dissected ovaries was removed by an ≈2-hr digestion with 1 mg/ml collagenase A, and oocytes were induced to mature with progesterone as described [59]. For capped mRNA microinjection experiments, the usual injected volume was 20 to 40 μ l per oocyte. *In vitro* fertilization was carried out as described [60]. For protein extracts, oocytes were homogenized at 4°C (5 μl per oocyte) in XB buffer: 10 mM K-HEPES pH 7.7, 100 mM KCl, 1 mM MgCl_2_, 50 mM sucrose, supplemented with 1× protease inhibitor cocktail (Roche Diagnostics) and 0.5 mM DTT and, where indicated, with a phosphatase inhibitor cocktail: 50 mM NaF, 10 mM β-glycerophosphate, 1 mM Na_3_VO_4_, 1 μ 1microcystin or 2 μorokadaic acid. The homogenates were then centrifuged at 12,000 g for 3 min at 4°C. MII-arrested egg extracts were prepared from unfertilized *Xenopus* eggs as described [61]. For phosphatase treatment, lambda protein phosphatase was added at 20 units/μ For phosphatase treatment, lambd°X.

### Antibodies

Rabbits were handled in the animal house of the Institut Universitaire de Technologie de Montpellier, which has an institutional agreement (number D34-172-8) from the Direction Départementale de la Protection des Populations (DDPP) de l’Hérault (France), which operates under the supervision of the Ministry of Agriculture and is dedicated to rabbit immunization and blood sampling. The protocol was not further submitted to the approval of an ethics committee as such approval was not necessary for these experiments under French and European legislation at the time they were conducted. Rabbits were kept under in the highest quality animal facilities, with all efforts taken to keep any suffering to an absolute minimum. At the end of the immunization protocol, rabbits were anaesthetized with Pentobarbital (30 mg/kg) and then sacrificed by intracardiac injection of Dolethal (Vetoquinol S.A.) 1.1 ml/kg. Polyclonal anti-Fbw7 and anti-pS18 antibodies were produced by immunizing rabbits with a synthetic peptide (MKRKLDHGSEVRSFS) corresponding to the N-terminal sequence common to the three *Xenopus* or human Fbw7 isoforms, and with a synthetic peptide (amino-acids 11 to 22) corresponding to the human Fbw7αor human Fbw7 isoforms, and with a synthetic peptide (amino-aKRRRTGGpSLRGN), respectively. Peptides were coupled to thyroglobulin for immunization and to bovine serum albumin (BSA) for affinity purification performed on nitrocellulose blots, as described [57]. For purification of anti-pS18 antibodies, immune sera were first depleted from the antibodies recognizing the non-phosphorylated sequence using immobilized BSA-CKRRRTGGSLRGN. Rabbit polyclonal antibodies against *Xenopus* cyclin E were previously described [62]. Rabbit *Xenopus* Cdc6 antiserum was a gift from M. Méchali (IGH, Montpellier, France); anti-CDC27 was a gift from T. Lorca (CRBM, Montpellier, France). Antibodies against hCyclin E (sc-247) and hCyclin B (sc-245) were purchased from Santa Cruz Biotechnology. Anti-ubiquitin antibodies were from Zymed (Thermo Fisher Scientific). Antibodies against α-tubulin (clone DM1A), hCyclin A (C4710), and anti-Flag M2 (F1804) were from Sigma-Aldrich. Phospho-(Ser)PKC substrate antibodies (2261) were from Cell Signaling, anti-HA (clone 12CA5) from Roche Diagnostics, and anti-GFP (TP401) from Torrey Pines Biolabs.

### Recombinant proteins

*x*Fbw7α DNA was amplified from pCS2-xFbw7α by PCR, and the product cloned into the *Eco*R1-*Not*1 sites of pGEX-4T-1. Site-specific mutations were generated using the QuikChange site-directed mutagenesis kit (Agilent Technologies). Expression of the GST-tagged xFbw7α (wild-type or S18A mutant), in *E*. *coli* BL21 cells was induced by 0.5 mM IPTG for 3 hr at 37°C. Cell pellets were resuspended in lysis buffer (50 mM MES pH 6.0, 150 mM NaCl, 20 mM EDTA, 0.5% Tween-20 supplemented with 1× protease inhibitor cocktail and 5 mM DTT), and sonicated in ice six times for 10 s each. Lysates were spun at 12,000 x g for 30 min and the supernatant incubated at 4°C with MagneGST^™^ glutathione beads (Promega) for 2 hr. Beads were collected with a magnet and washed with lysis buffer supplemented with 500 mM NaCl for storage at 4°C. Recombinant baculovirus expressing *Xenopus* cyclin E with a GST tag at its N-terminus was generated using the pFastBac-GST2 system. Baculovirus-encoded GST-cyclin E was expressed in Sf9 insect cells seeded into 75 cm^2^ dishes at 30–40% confluence and infected at a MOI of 0.1 for 2 days at 27°C. Cell pellets were resuspended in lysis buffer (50 mM Tris pH 7.5, 500 mM NaCl, 1 mM EDTA, 1 mM EGTA, 0.5% Tween-20, 50 mM NaF, 10 mM β-glycerophosphate, 1 mM Na_3_VO_4_, 1 μ 1microcystin, 1 mM DTT and 1× protease inhibitor cocktail). GST-cyclin E was affinity purified with MagneGST^™^ glutathione beads. Recombinant Cdc25B was purified under native conditions as described previously [63]. *In vitro* transcription and translation in rabbit reticulocyte lysate was performed using the TNT system (Promega).

### *In vitro* kinase assays

For the PKCα kinase assay, 3 μkinase assay, α (wild-type or S18A mutant) pre-bound to MagneGST^™^ glutathione beads equilibrated in kinase buffer (20 mM Tris pH 7.5, 100 μpH 7.5, 10mM MgAc, 1 mM DTT) were incubated with 30 ng of recombinant PKCα (Thermo Fisher Scientific) in the presence of activators (750 μ(Therm_2_, 25 μ2phosphatidyl serine and 0.8 μsediolin) or in the presence of 0.5 mM EGTA as a negative control. Samples were incubated for 5 min at 30°C, denatured by adding SDS sample buffer, and processed for SDS-PAGE and immunoblotting analysis. To perform *in vitro* kinase assays in MII-arrested egg extract, 0.5 to 1 μxtract, 0.5 toα (wild type or S18A mutant) pre-bound to MagneGST^™^ glutathione beads equilibrated in XB-EGTA buffer supplemented with 7 mM EGTA were added to 100 μEGTA were added to 100 0 ated in XB-EGTA buffer supplemented with 7, denamM DTT. Assays were started by the addition of 5 μCi of [γ-^33^P] ATP and incubated at 23°C for 30 min. When indicated, the cPKC inhibitor Gö6976 (Sigma-Aldrich) was added at a final concentration of 1 μinhibitor Gö6976 (Sigma-Aldrich) was α beads. Beads were collected and washed five times with XB buffer supplemented with 500 mM NaCl and 1% Triton X-100, then twice with XB buffer supplemented with 1% Triton X-100 at 4°C. SDS sample buffer was added and samples were boiled and processed for SDS-PAGE, western blotting and phosphorimaging analysis.

### Cell synchronization and transient transfection

HeLa cells (American Type Culture Collection) were maintained in DMEM with 10% FBS. HeLa cells were arrested at the G1/S phase transition by addition of 2.5 mM thymidine (Sigma-Aldrich) for two blocks of 24 hr separated by an interval of 12 hr, and in pro-metaphase with 200 ng/ml nocodazole (Sigma-Aldrich) for 14 hr. Mitotic cells were recovered by shake-off when indicated. Cells were transfected with PKCα-pEGFP-N1, PKC δ-pEGFP-N1, PKCε-pEGFP-N1, PKCβ-pEGFP-N1 constructs [64] and with pCS2-FLAG-Fbw7α or pCS2-HA-Fbw7α using GeneCellin^™^ (BioCellChallenge), following the manufacturer’s instructions, except that we used no more than 2 to 3 μg of pCS2-FLAG-FBw7α and/or pHA-Fbw7α in 10 cm dishes. For cycloheximide chase experiments, cells were treated with cycloheximide (50 μg/ml) for the indicated periods of time. Harvested cells were lysed in SDS sample buffer and processed for immunoblot analysis.

### Immunoprecipitation

**Fig 1:**
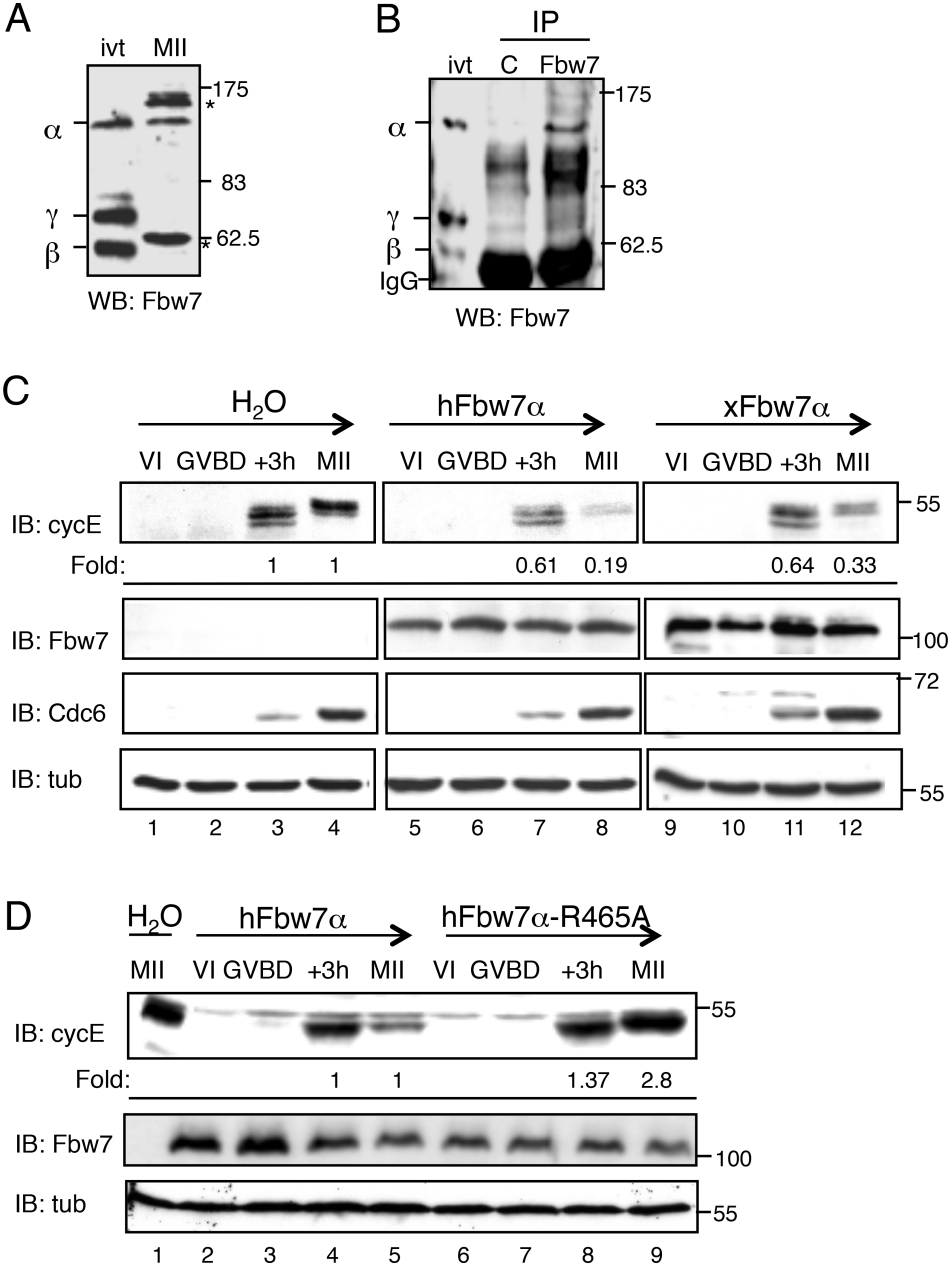
xFbw7α protein is expressed and xCyclin E can be degraded in an Fbw7α-dependent manner in MII-arrested eggs. A. Immunoblot analysis with anti-Fbw7 antibodies of protein extracts from MII-arrested oocytes. ivt: xFbw7α, β and γ isoforms translated *in vitro*. Asterisks denote non-specific immunoreactive bands. B. MII-arrested egg extracts immunoprecipitated with control IgG (C), or anti-Fbw7 antibodies. C. Stage VI oocytes were microinjected with mRNAs coding for hFbw7α or xFbw7α, or with H_2_O as a control. 12 hr later, they were induced to mature by addition of progesterone. The oocytes were collected at GVBD, 3 hr after GVBD (+3h) and during metaphase II arrest (MII). The equivalent of two oocytes was loaded in each lane. Oocyte extracts were analyzed with antibodies directed against Fbw7, xcyclin E, xCdc6 or tubulin. Cyclin E levels were quantified using ImageJ software and were normalized to tubulin. D. Immunoblot analysis of progesterone-treated oocytes microinjected with hFbw7α or hFbw7α-R465A mRNAs and collected during meiotic maturation.

Endogenous Fbw7α was immunoprecipitated at 4°C for 20 min from MII-arrested egg extracts (50 μl) or for 90 min from 500 μg of proteins from HeLa cell lysates using polyclonal anti-Fbw7 antibodies and protein A magnetic Dynabeads (Thermo Fisher Scientific). After several washes with XB buffer, bead pellets were boiled in SDS sample buffer, separated by 7% SDS-PAGE and transferred onto Immobilon-P membranes (Millipore). Endogenous Fbw7α was visualized with anti-Fbw7 or anti-pS18 antibodies and HRP-conjugated protein A (Thermo Fisher Scientific) was used as a second step, except in Fig 1B, where TrueBlot^®^ anti-rabbit Ig IP beads (eBioscience) were used. For co-immunoprecipitation of ectopically expressed proteins, HeLa cells were co-transfected with the corresponding constructs. Cells were homogenized in lysis buffer: 50 mM K-HEPES pH 7.7, 100 mM KCl, 1 mM EDTA, 1% Triton X-100, 1 mM DTT, 10% glycerol, 1× protease inhibitor and phosphatase inhibitor cocktail (Roche Diagnostics) and 25 μM MG132 (BML-PI102-0005, Enzo Life Sciences) at 4°C for 30 min. After centrifugation at 12,000 g for 20 min, 2 μg anti-HA or anti-FLAG antibodies were added to the pre-cleared supernatants. Ectopic Fbw7α proteins were immunoprecipitated at 4°C for 1 hr from 500 μg to 1 mg of protein cell lysates and collected on protein-A or -G magnetic beads.

### Indirect immunofluorescence microscopy

Cells grown on coverslips were fixed with 3.7% paraformaldehyde, then permeabilized with 0.25% Triton X-100 and cold methanol at -20°C. Cells were washed with PBS and blocked with 1% FCS/PBS for 15 min. Incubation with primary antibodies was carried out at 37°C for 1 hr and with secondary antibodies at room temperature for 40 min. DNA was stained with DAPI (D9542, Sigma-Aldrich). Microscopic examinations were performed with a Leica DM 6000 microscope, using a 63×/1.4 NA oil immersion objective lens. Photomicrographs were captured with a 12-bit CoolSNAP HQ2 1 camera. Images were acquired as TIFF files using the MetaMorph imaging software (Molecular Devices).

## Results

### Fbw7α is expressed in *Xenopus* eggs

As we wished to investigate the mechanism by which cyclin E can accumulate in a mitotic-like phase, our first question was to determine whether the Fbw7 isoforms are expressed in eggs (S1A Fig). Using semi-quantitative PCR we detected equal amounts of Fbw7α and -β transcripts in immature oocytes and mature eggs, whereas Fbw7γ transcripts were not detected (S1B Fig). We raised an antibody against a peptide corresponding to the N-terminal sequence common to the three Fbw7 isoforms (S2A Fig), and used this to establish by immunoblotting which of the three isoforms are present in mature oocytes. Only one band, which comigrated exactly with *in vitro* translated *Xenopus* Fbw7α (xFbw7α, 110 kDa), was recognized by our antibody (Fig 1A). Using this antibody we were able to specifically immunoprecipitate this protein from metaphase II (MII)-arrested egg extracts (Fig 1B). We thus concluded that at least the Fbw7α isoform is expressed in MII-arrested eggs and that the stability of cyclin E cannot be explained by the absence of Fbw7.

### Cyclin E can be degraded in an Fbw7α-dependent manner in eggs

As cyclin E capture by Fbw7 requires its phosphorylation within one or two specific phosphodegron motifs [4, 5, 65], it was important to check whether its phosphorylation status in eggs is compatible with its Fbw7-mediated degradation. Human (hFbw7α) or *Xenopus* xFbw7α mRNAs were microinjected into stage VI arrested oocytes that were then induced to mature by addition of progesterone (Fig 1C). As shown in control (H_2_O-injected) oocytes, slower migrating, i.e. phosphorylated, forms of cyclin E were readily detectable 3 hr following germinal vesicle breakdown (GVBD) and completion of the first meiotic division. Hyper-phosphorylated forms of cyclin E accumulated subsequently in mature oocytes arrested in metaphase II, as previously reported [58]. In contrast, expression of either hFbw7α or xFbw7α caused a drastic decrease (>70%) of the level of hyperphosphorylated cyclin E (Fig 1C, compare lanes 4, 8 and 12) in mature oocytes without impacting on their maturation, as demonstrated by the timing of Cdc6 expression (Fig 1D). Furthermore, expression of an Fbw7α mutant (hFbw7-R465A) that cannot bind cyclin E [43] had no effect on the accumulation of phosphorylated cyclin E (Fig 1D, compare lanes 5 and 9). We thus concluded that cyclin E is phosphorylated in a manner appropriate for its Fbw7α-dependent degradation, and that therefore its stability must be due to the inactivation of endogenous Fbw7α.

### PKC-dependent phosphorylation of Fbw7α in MII egg extracts

**Fig 2:**
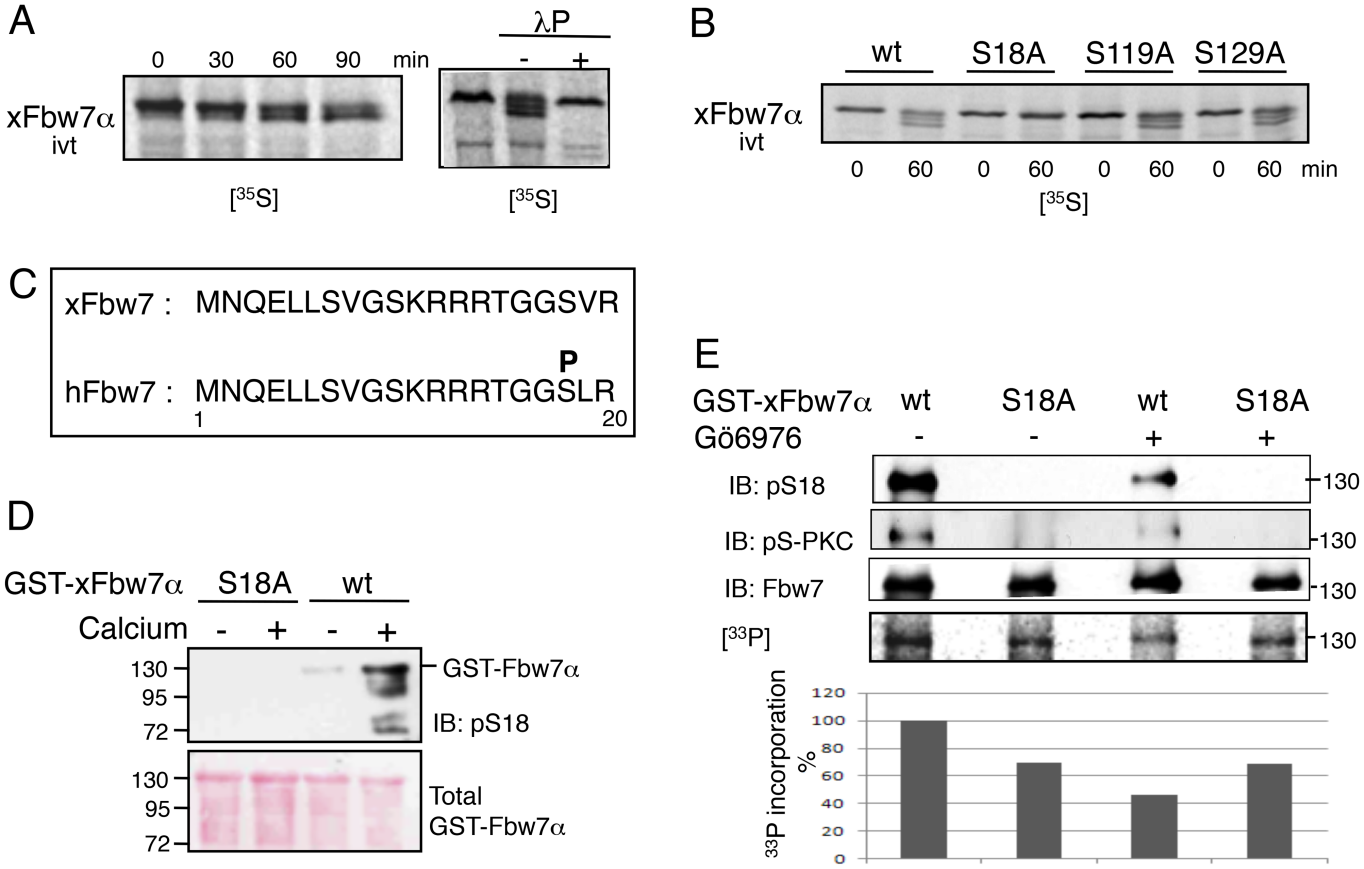
xFbw7α is phosphorylated by PKC on S18. A. *In vitro* translated [^35^S]-xFbw7α was incubated in MII-egg extracts prepared with phosphatase inhibitors (left panel). [^35^S]-xFbw7α-wt was incubated 1 hr in MII-egg extracts prepared with phosphatase inhibitors and an equal amount of sample was subsequently incubated with an excess of lambda protein phosphatase (λP) and analyzed by phosphorimaging (right panel). (B) *In vitro* translated [^35^S]-xFbw7α-wt, -S18A, -S119A or -129A were incubated 1 hr in MII-egg extracts. C. Sequence alignment of the Fbw7α N-terminal region from *Xenopus* and human. D. GST-xFbw7α-wt or -S18A bound to magnetic beads were incubated with PKCα, activated (+) or not (-) and then submitted to immunoblot analysis with anti-pS18 antibodies. Total GST-Fbw7 was detected by Ponceau S staining. (E) GST-xFbw7α wt or S18A were incubated in MII-egg extracts plus phosphatase inhibitors, supplemented (+) or not (-) with Gö6976 and with [γ-^33^P]-ATP for 1 hr at 23°C and analyzed by immunoblotting and by phosphorimaging for quantification of ^33^P incorporation (lower panel).

Our antibodies against Fbw7 failed to recognize the endogenous xFbw7 in egg extracts prepared with extraction buffer containing phosphatase inhibitors (Fig 1C, see H_2_O-injected oocytes lane MII, and S2B Fig), suggesting that the recognition epitope encompasses a site for phosphorylation. We thus analyzed the behavior of [^35^S]-Met-labeled xFbw7α produced in rabbit reticulocyte lysates, following its addition in egg extracts. Time course experiments showed that surprisingly, the radiolabeled xFbw7α was converted to forms exhibiting increased electrophoretic mobility. Treatment of the extract with λ-phosphatase abolished this accelerated migration (Fig 2A), demonstrating that this electrophoretic migration is indeed due to phosphorylation, as seen for certain proteins including Cdk2 [66]. Since only Fbw7α, and not the β and γ isoforms, presented this accelerated mobility, we hypothesized that the phosphorylation site(s) lie within the N-terminal part specific for Fbw7α. We inspected this region for S/T residues present within putative phosphorylation motifs and identified several serine residues including S18, S119 and S129. Fbw7α constructs in which each of these serines was individually substituted for alanine were generated, and their migration properties were tested in *Xenopus* egg extracts. Fbw7α-S18A was the only mutant unable to migrate as wild-type (wt) Fbw7α (Fig 2B), showing that Fbw7α is phosphorylated on this residue in eggs. This site was previously shown to be phosphorylated by PKC in mammalian cells. To analyze the phosphorylation of this site in our system, we generated an antibody against a peptide derived from Fbw7, containing phosphoserine at residue 18 (pS18) (Fig 2C). To validate the specificity of our pS18 antibody, and to confirm that PKC can directly phosphorylate Fbw7, we performed an *in vitro* kinase assay in which recombinant GST-xFbw7α-wt or -S18A proteins were incubated with purified PKCα in the presence or absence of activating Ca^2+^. A specific pS18 signal was detected with xFbw7α-wt but not with the S18A mutant, and this signal was over ten-fold stronger in the presence of Ca^2+^ (Fig 2D). Taken together, these data are consistent with our pS18 antibody being specific for Fbw7α only when phosphorylated on S18, and with the direct phosphorylation of Fbw7α on S18 by PKCα. Furthermore, we performed a radiolabeled kinase assay by incubating recombinant Fbw7α-wt or -S18A in MII extracts in the presence of [*γ-*^33^P] ATP, supplemented or not with the PKC inhibitor Gö6976 [67]. As shown in Fig 2E, while addition of Gö6976 had no significant impact on the [^33^P] labeling of Fbw7α-S18A, it severely reduced that of Fbw7α-wt by more than 50%. This, together with the observation that the anti-phospho-PKC-substrate signal was significantly reduced by Gö6976 and abolished by the S18A mutation, strongly supports the notion that Ser18 is the major PKC target site in Fbw7α (Fig 2E). Furthermore, since the S18A mutation reduced Fbw7α phosphorylation by 30% in the absence of PKC inhibitor, Ser18 also appears to be a major site for Fbw7α phosphorylation in MII egg extracts.

### Phosphorylation of endogenous xFbw7α by PKC prevents cyclin E ubiquitylation

**Fig 3:**
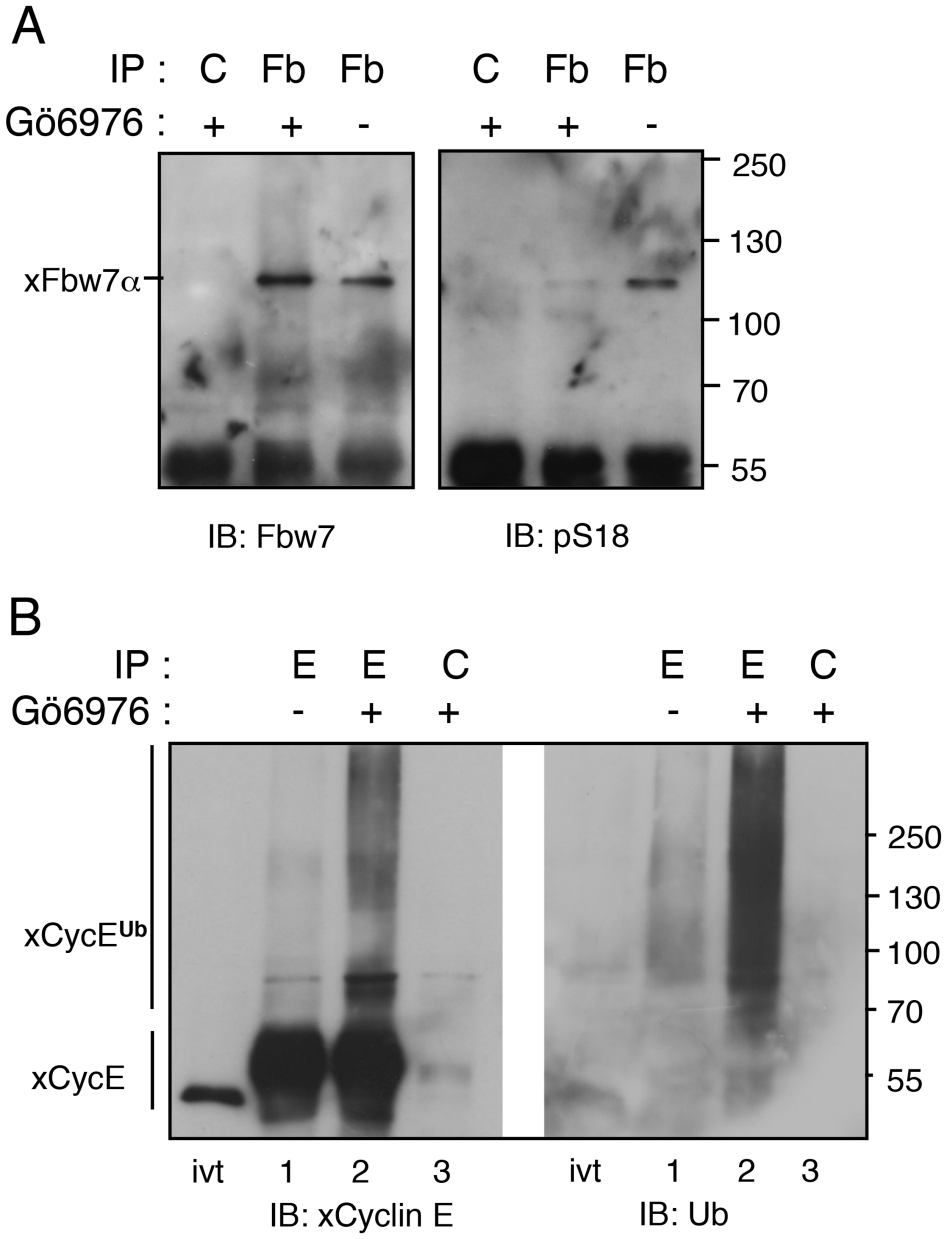
Dephosphorylation of Ser18-xFbw7α correlates with cyclin E ubiquitylation. A. *Xenopus* MII-arrested egg extracts were treated or not with Gö6976 and subjected to immunoprecipitation with anti-Fbw7 (Fb) or control antibodies (C) and analyzed by immunoblotting to detect xFbw7α and phosphorylated Ser18 (pS18). B. The same extracts were subjected to immunoprecipitation with anti-xCyclin E (E) or control antibodies (C) and analyzed by immunoblotting with anti-xCyclin E and anti-ubiquitin antibodies.

To address the putative involvement of PKC in the inactivation of Fbw7, we next examined how the stability of cyclin E in egg extracts correlates with the phosphorylation status of endogenous xFbw7-Ser18. Egg extraction buffer was supplemented or not with the PKC inhibitor Gö6976 (phosphatase inhibitors were omitted), and endogenous Fbw7 or cyclin E were immunoprecipitated for only 20 min from the resulting extracts with antibodies against Fbw7 or cyclin E. Both the anti-Fbw7 and anti-pS18 antibodies recognized endogenous xFbw7 without the PKC inhibitor, while the anti-pS18 signal was no longer detected in the precipitate from Gö6976-treated extracts, confirming that the endogenous xFbw7 is phosphorylated by PKC on Ser18 (Fig 3A). Importantly, the non-phosphorylation of Fbw7 at S18 caused by the PKC inhibitor correlated with the occurrence of a significant ubiquitylation of cyclin E precipitates (Fig 3B), suggesting that when Fbw7 is kept phosphorylated on Ser18, it is inactive towards its substrate cyclin E.

### Phosphorylation of human Fbw7α on S18 occurs during mitosis

**Fig 4:**
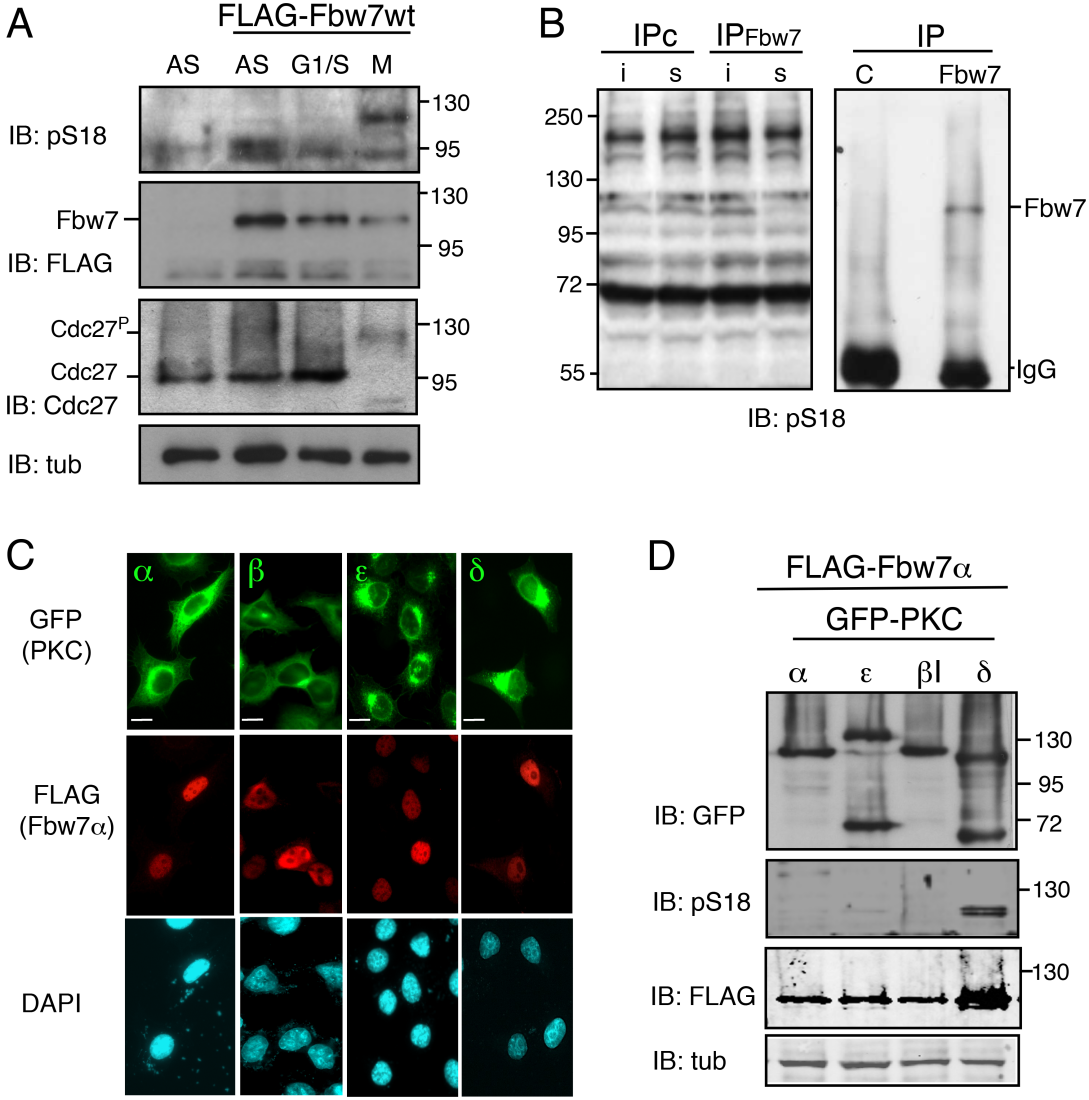
Endogenous and ectopic Fbw7α are phosphorylated at Ser18 in M-phase. A. FLAG-Fbw7α was transfected or not in HeLa cells, as indicated. Total extracts of asynchronous cells (AS), cells synchronized in G1/S or arrested in pro-metaphase and recovered by shake-off (M) were analyzed by SDS-PAGE and immunoprobed for pS18-Fbw7α and FLAG-Fbw7α. The migration of Cdc27 was used as a marker of M phase (Cdc27^P^), and anti-tubulin as a loading control. B. HeLa cell extracts (500 μg) from cells arrested in prometaphase were subjected to immunoprecipitation with anti-Fbw7 or control antibodies and analyzed by immunoblotting to detect phosphorylated Ser18 (pS18). Input 10% (i), supernatant after IP (s). C. HeLa cells grown on coverslips were co-transfected with FLAG-Fbw7α and either GFP- PKCα, PKCβ1, PKCε or PKCδ, as indicated. Coverslips were fixed and stained with FLAG antibodies and DAPI. Scale bar, 10 μm. D. HeLa cells were co-transfected with FLAG-Fbw7α and the different GFP-PKC constructs, as indicated. At 24 hr post-tranfection, cells were harvested and the levels of GFP-PKC, pS18-Fbw7α, FLAG-Fbw7α and tubulin were monitored by immunoblotting.

Having highlighted the PKC-dependent phosphorylation and inactivation of Fbw7 in the *Xenopus* egg in metaphase II, i.e. in the absence of a nuclear envelope, we next wondered whether this regulatory mechanism is conserved during somatic cell cycles. To this end we analyzed Fbw7α phosphorylation at different cell cycle phases. FLAG-Fbw7α was expressed in HeLa cells, then extracts of these cells blocked either at the G1/S phase transition or in prometaphase were immunoprobed with the anti-pS18 antibodies. Under these conditions, a signal was obtained only in cells blocked in prometaphase (Fig 4A). We also analyzed the phosphorylation status of the endogenous Fbw7α in HeLa cells blocked in prometaphase. A faint band, specifically depleted from the whole cell extract with the Fbw7 antibodies, was recovered and recognized by the pS18 antibodies on Fbw7 immunoprecipitates (Fig 4B), indicating that Fbw7α phosphorylation by PKC likely occurs in M-phase.

To provide complementary evidence that PKC phosphorylates Fbw7α in M-phase, we examined their respective subcellular localization. Although PKCs are generally cytoplasmic kinases, among the 11 isoenzymes of the PKC family [68], one isoenzyme, PKCδ, was reported to contain a nuclear localization signal [69], and to localize in the nucleus under certain circumstances [70]. We thus co-transfected HeLa cells with FLAG-Fbw7α and either GFP- PKCα, PKCβ1, PKCε or PKCδ. While Fbw7α was nucleoplasmic in the four assays, PKCδ was the only isoform that localized both in the cytoplasm and in the nucleus (Fig 4C). Of note, to avoid a mislocalization of Fbw7α in the cytoplasm, a low amount of plasmid DNA was used in tranfections. Consistent with their respective subcellular localizations, phosphorylation of FLAG-Fbw7α on S18 was detected when Fbw7α was co-expressed with PKCδ and not with the cytoplasmic PKC-α, β1 or ε isoenzymes (Fig 4D).. Together these results indicate that under physiological conditions PKC-mediated phosphorylation of Fbw7 at Ser18 may be restricted to the mitotic phase in cycling somatic cells, occurring when both proteins meet upon nuclear envelope breakdown.

### A negative charge at S18 increases Fbw7α stability itself

**Fig 5:**
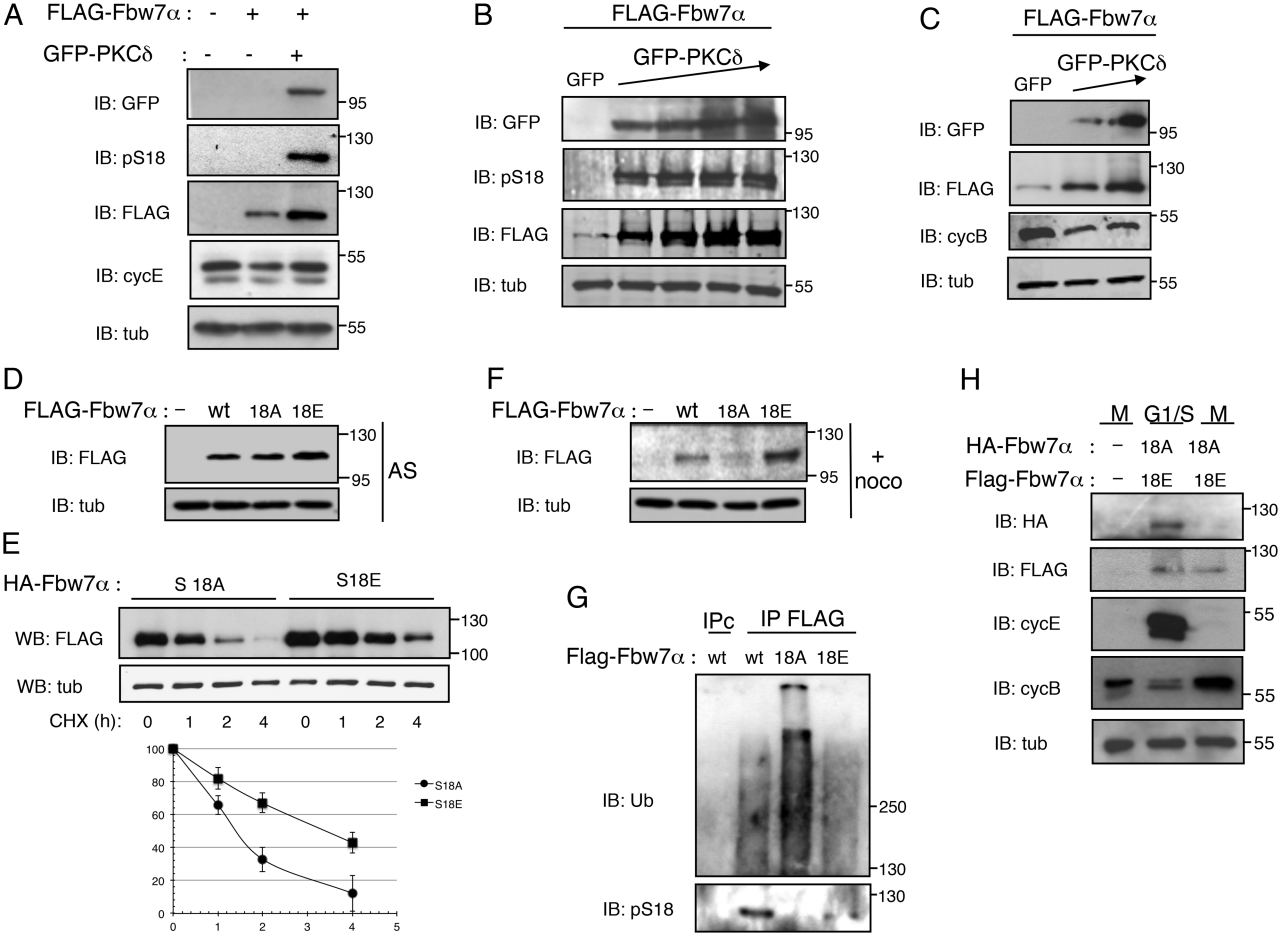
PKC phosphorylation stabilizes Fbw7α in mitosis. A. HeLa cells were co-transfected with GFP-PKCδ and FLAG-Fbw7α. Cell extracts were probed for GFP-PKCδ, pS18-Fbw7α, FLAG-Fbw7α, cyclin E and tubulin. B. HeLa cells were co-transfected with FLAG-Fbw7α and either GFP or increasing amounts of GFP-PKCδ and analyzed by immunoblotting, as indicated. C. HeLa cells, co-transfected as in B, were treated with nocodazole and probed for cyclin B to monitor progression into M phase. D. HeLa cells were transfected or not (-) with Flag-Fbw7α-wt, -S18A or -S18E, and Fbw7α levels were analyzed by immunoblotting from asynchronous (AS). E. HeLa cells were transfected with FLAG-Fbw7α-S18A or FLAG-Fbw7α-S18E, treated with cycloheximide (CHX) for the time indicated and analyzed by immunoblotting (upper panel). Fbw7 protein levels of two independent experiments were quantified with ImageJ software and normalized to tubulin. The results were plotted as the relative Fbw7 levels compared to those at the time 0 of CHX treatment (lower panel). F. HeLa cells were transfected as in D and treated with nocodazole (+ noco). G. Extracts from HeLa cells, transfected and treated with nocodazole as in F were supplemented with MG132 and phosphatase inhibitors to be subjected to immunoprecipitations with anti-FLAG and control antibodies (IPc) and analyzed by immunoblotting to detect the presence of ubiquitin and pS18-Fbw7α. H. HeLa cells, co-transfected or not (-) with FLAG-Fbw7α-S18E and HA-Fbw7α-S18A, were synchronized in G1/S or arrested in prometaphase and recovered by shake-off. Cell extracts were analyzed by immunoblotting to detect the presence of Fbw7α. Anti-cyclin E and anti-cyclin B were used as markers of G1/S and M-phase respectively.

In the *Xenopus* egg, PKC phosphorylation of Fbw7α at S18 correlated with the stability of its substrate, cyclin E (Fig 3B). To determine whether this is also the case in human cells, the PKCδ isoenzyme was used in order to examine how overexpression of a PKC affects the level of endogenous cyclin E. Whereas expression of FLAG-Fbw7α decreased the amount of cyclin E by 30%, co-expression of FLAG-Fbw7α with GFP-PKCδ completely restored the steady-state level of cyclin E (Fig 5A), suggesting that, as in *Xenopus* eggs, PKC-dependent phosphorylation of Fbw7α leads to its inactivation. More importantly, phosphorylation at S18 also correlated with a large increase of Fbw7 levels (Figs 4B and 5A). In fact, the extent of PKCδ-dependent S18 phosphorylation tightly correlated with the accumulation of Fbw7α and particularly of forms exhibiting accelerated mobility as observed in *Xenopus* egg extracts (Fig 5B). These results indicate that the PKC-dependent phosphorylation of Fbw7α at Ser18 not only protects cyclin E but also stabilizes Fbw7 itself.

Since, as reported in previous studies [71], overexpression of PKCδ affected cell cycle progression as shown in Fig 5C by the decrease of cyclin B levels, we monitored the impact of a negative charge on the residue in position 18 by monitoring the expression levels of wild-type Fbw7α and its phospho-deficient S18A and phospho-mimetic S18E mutant forms. In asynchronous cells, using equal low amounts of FLAG-Fbw7α expression plasmids, Fbw7α-S18E was reproducibly expressed in slightly higher amounts than Fbw7α-wt or Fbw7α-S18A (Fig 5D), suggesting that the phospho-mimetic mutant is the most stable mutant form. Indeed, the half-life of Fbw7α-S18E was prolonged compared to Fbw7α-S18A (Fig 5E). The difference was however much more obvious in prometaphase-arrested cells. Strikingly, Fbw7α-S18A was no longer detected, while the steady-state abundance of Fbw7α-S18E was higher than that of wild-type Fbw7α (Fig 5F). Notably, Fbw7α-S18A immunoprecipitates from cell extracts arrested in prometaphase and treated with the proteasome inhibitor MG132 displayed a strong ubiquitin smear compared to Fbw7α-wt phosphorylated at S18 or Fbw7α-S18A (Fig 5G), demonstrating that when Fbw7α is either phosphorylated or harbors a negative charge on residue 18, it is not subjected to self-ubiquitylation. To further support the direct link between Fbw7α stabilization and the presence of a negative charge at Ser18, equal low amounts of FLAG-Fbw7α-S18E and HA-Fbw7α-S18A expression plasmids were co-transfected in cells arrested at the G1/S phase transition or in prometaphase. While both mutants were detected in S phase, the phospho-mimetic mutant was the only one still detected in mitosis (Fig 5H). Taken together, these results demonstrate that a phosphorylation-dependent event at Ser18 is required to stabilize Fbw7α in mitosis.

### A negative charge at S18 reduces the capacity of Fbw7 to dimerize and to bind cyclin E

**Fig 6:**
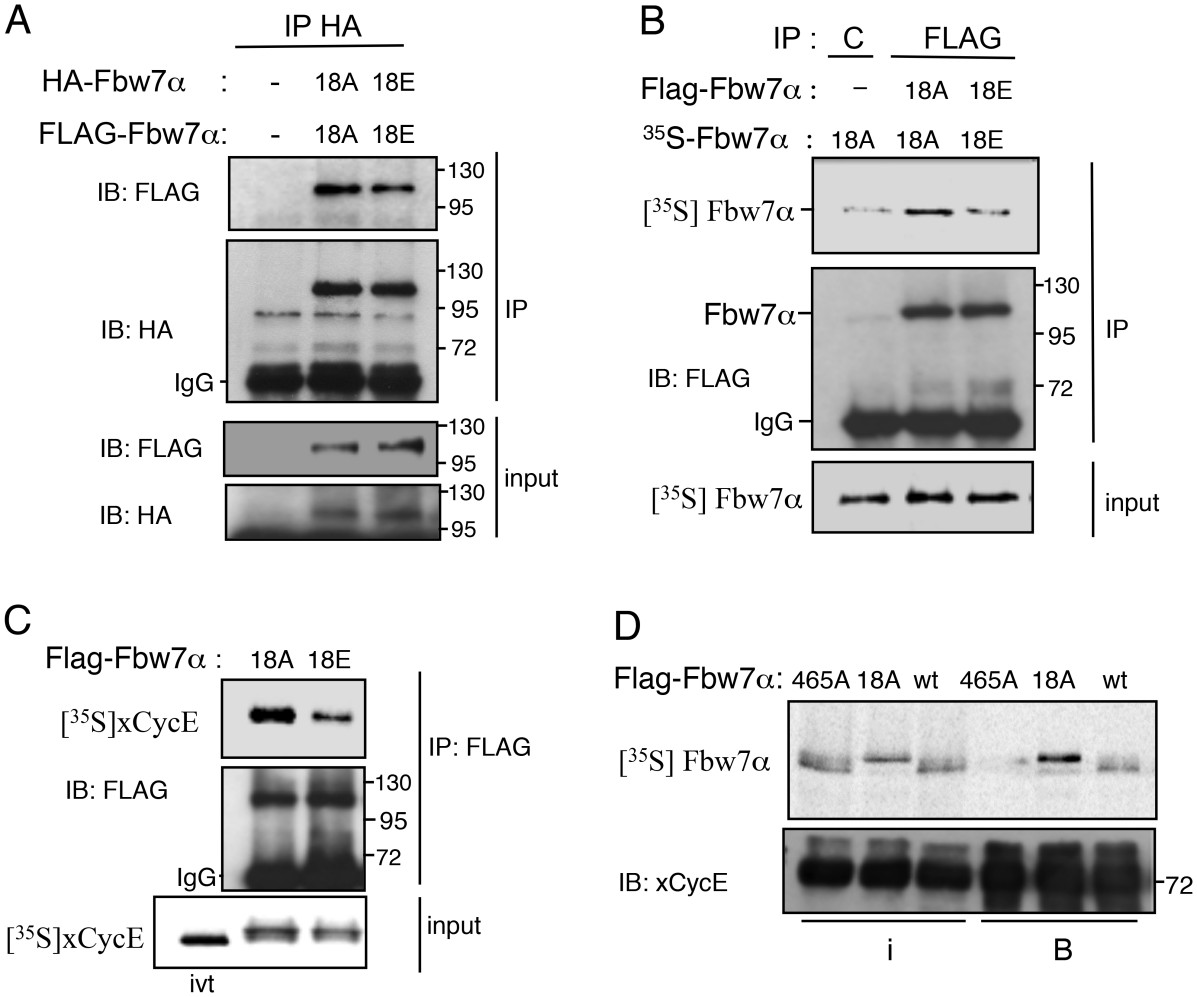
Fbw7 dimerization and cyclin E binding is reduced by a negative charge at Ser18. A. FLAG-and HA-tagged versions of Fbw7α either S18A or S18E were co-transfected in HeLa cells and analyzed for their interaction by immunoprecipitation with HA antibodies. Co-precipitated FLAG-tagged Fbw7α was detected by immunoblotting with an anti-FLAG antibody. The membrane was re-probed with HA antibodies. B. Fbw7 or control immunoprecipitates from HeLa cells transfected or not (-) with FLAG-Fbw7α-S18A or –S18E were mixed with *in vitro* translated [^35^S]-Fbw7α-18A or -18E (with no tag in N-terminal), as indicated. Complexes were analyzed by phosphorimaging and immunoblotting. C. *In vitro* translated [^35^S]-xCyclin E was first incubated with a recombinant Cdc25B phosphatase to activate its associated Cdk2 partner from the reticulocyte lysate and further incubated in MII-egg extracts with phosphatase inhibitors for 30 min. Phosphorylated cyclin E was mixed with FLAG-hFbw7α-18A or-18E immunoprecipitates. Complexes were analyzed by phosphorimaging and immunoblotting. D. *In vitro* translated [^35^S]-FLAG-hFbw7α-465A, -18A or -wt were incubated in MII-egg extracts with phosphatase inhibitors for 60 min and mixed with GST-cyclin E bound to magnetic beads. The mutant hFbw7α-465A that cannot bind cyclin E serves as a negative control. Input represents 10% of the total extract (i), total beads (B). Complexes were analyzed by phosphorimaging and immunoblotting.

Next, we went on to explore how a negative charge at Ser18 might improve Fbw7α stability while preventing cyclin E ubiquitylation. We found that the known interactions of Fbw7, namely with the SCF component Skp1 and with ubiquitin or the deubiquitinase USP28, were not affected by the charge at position 18 (S3A–S3C Fig). A recent study revealed that Fbw7 exists as dimers to optimize substrate binding and importantly that ectopically expressed Fbw7 monomers are stable [44]. This prompted us to test the putative involvement of S18 phosphorylation in Fbw7α dimerization. We addressed this issue by co-transfecting HeLa cells with HA-Fbw7α and FLAG-Fbw7α either S18A or S18E, and analyzing their interaction using immunoprecipitation. Interestingly, we observed that the negative charge at Ser18 reduced co-precipitation of FLAG-Fbw7α with HA-Fbw7α by 40% (Fig 6A). We also performed *in vitro* binding studies by mixing FLAG-Fbw7α immunoprecipitates with radiolabeled Fbw7α produced in rabbit reticulocyte lysates by *in vitro* transcription-translation. The intensity of the Fbw7α-S18E radioactive band retained on Fbw7α-S18E precipitates was reduced by 80% compared to the reaction performed with the Fbw7α-S18A proteins (Fig 6B), demonstrating that addition of a negative charge at S18 interferes with Fbw7α dimerization. Consistent with this conclusion and knowing that dimerization of Fbw7 is important for cyclin E binding, we further found that the interaction between radiolabeled cyclin E, phosphorylated in egg extracts, and the Fbw7α-S18E mutant form was also severely reduced compared with the Fbw7α-S18A mutant which forms dimers (Fig 6C). Conversely, radiolabeled Fbw7α-wt phosphorylated in egg extracts was retained at a much smaller amount on GST-cyclin E beads compared to the Fbw7α-S18A mutant form (Fig 6D). Taken together, these data support the notion that PKC negatively regulates cyclin E binding by interfering with Fbw7α dimerization.

## Discussion

The present study sheds light on an unexpected role for PKC in regulating SCF-Fbw7α activity in an M-phase-specific manner. Our results show that once phosphorylated by PKC, Fbw7α is inactive towards its substrate cyclin E and is itself stabilized, thus, kept in a resting inactive state.

The Fbw7α site targeted by PKC, Ser18, was previously shown to be phosphorylated by various PKC isoenzymes *in vitro* and in mammalian cells. However, no functional role for S18 phosphorylation was identified [51]. As we initially identified S18 as a target of PKC in *Xenopus* eggs arrested in metaphase II of meiosis, we analyzed this phosphorylation event in different cell-cycle phases in mammalian cells. In order to not affect cell-cycle progression, we had to greatly decrease the amount of Fbw7α-expressing vector used in transfection assays. As a consequence, the subcellular localization of Fbw7α was mainly in the nucleoplasm during interphase. This allowed us to demonstrate that because PKCs reside in the cytoplasm, they can only target Fbw7α during M-phase, i.e., when the nuclear envelope breaks down.

*Xenopus* as well as mammalian eggs express many PKC isoenzymes belonging to each of the three groups, classified according to their structures and mechanism of activation: “conventional” cPKCs (PKCs α, βI, βII or γ), “novel” nPKCs (PKC δ, ε,η and θ) or “atypical” aPKCs (PKC ζ and ι/λ) [72–74]. In egg extracts, phosphorylation at S18 was efficiently compromised in the presence of 1 μM Gö6976, suggesting that this event might be dependent on the activity of a cPKC [67], however, at this concentration, this compound may lack specificity among members of the PKC family. Since the accumulation of cyclin E in mature eggs is canceled by expression of exogenous Fbw7α, whatever the PKC involved in S18 phosphorylation, its level is not sufficient to phosphorylate and inactivate most of the exogenous Fbw7α that was overexpressed during egg maturation. In addition, our data show that PKC is a limiting factor in HeLa cells, as only a low amount of exogenous Fbw7α-wt was phosphorylated at Ser18 and protected from degradation. Welcker *et al*. have shown that ectopic Fbw7α is unstable compared with endogenous Fbw7α [44]. This suggested that a limiting factor such as a deubiquitinase could not ensure the protection of an excess of Fbw7. However, they also demonstrated that exogenous monomers of Fbw7 are stable, most likely because Fbw7 degradation requires trans-ubiquitylation within a dimer. In the present study, we identify PKC as another limiting factor contributing to Fbw7α stability but whose protective effect appears to be linked to the decreased capacity of Fbw7α to form dimers once phosphorylated by PKC.

How does phosphorylation of S18 impede Fbw7α dimerization? To investigate the structural basis for this effect, we performed a computational analysis of the isoform-specific N-terminal sequence of Fbw7α. Of note, the available crystal structure of Fbw7 was obtained with a truncated protein (residues 263–707) [43]. The Fbw7α N-terminal specific sequence, 167 residues in length, which contains an unusually high proportion of negative charges (S1A Fig) was predicted to be intrinsically disordered by five disorder prediction methods (S4A Fig). This type of disordered region, highly flexible, generally harbors a variety of conformations that are in dynamic equilibrium. When S18 is not phosphorylated, whatever the adopted conformation, it does not affect the dimerization process of the α-isoform. Mechanistically, the phosphorylation of Fbw7α on S18 could create a new charge-charge interaction, triggering a disturbance that could impede the formation of dimers.

Although the regulation of Fbw7α by PKC is normally restricted to M-phase, an attractive hypothesis is that it could also operate in response to cellular stress. Consistent with this, we observed that ectopic expression of PKCδ induced stabilization of Fbw7α while impairing Fbw7α-mediated degradation of cyclin E. Translocation of PKCδ into the nucleus has been demonstrated after exposure of cells to various genotoxic stresses, and it is involved together with p53 in the apoptotic responses [70]. Our data therefore support the notion that maintaining adequate levels of Fbw7α in a resting inactive state must be important for a cell transiently arrested, such is the case for an egg awaiting fertilization or a cell awaiting its fate, that is, survival or apoptosis, following DNA damage. Such a regulation has the advantage of allowing transient inactivation of the protein, which in turn permits its activity to rapidly recover once the regulatory signal has stopped. In this regard, the fact that this transient phosphorylation event occurs within the disordered domain of Fbw7α is in agreement with the notion that these intrinsically disordered regions serve as excellent substrates to facilitate rapid conformational changes via post-translational modifications [75, 76].

Of note, Fbw7α is not the only target of PKC whose phosphorylation induces stabilization. PKC-mediated phosphorylation of p27^Kip1^ also promotes its accumulation, contributing to cell cycle arrest [77].

Under normal conditions, the evolutionarily conserved pathway highlighted in this study ensures the protection of Fbw7α upon nuclear envelope breakdown. However, as a consequence of this regulation, Fbw7α is not functional in M-phase. Thus, ubiquitylation of potential mitotic Fbw7 substrates should be assumed by another Fbw7 isoform. One identified mitotic substrate of Fbw7 is the anti-apoptotic protein Mcl1, which was shown to be targeted by Fbw7 during a prolonged mitotic arrest [6]. To date, one study has reported the involvement of the Fbw7β isoform in the degradation of Mcl1 [78]. The same may be true in response to various cellular stresses, as Fbw7β is the only p53-responsive isoform and as reported recently, the most potently induced p53 target gene in HCT116 cells [35]. Although Fbw7α is the most abundant isoform and is thought to perform most of Fbw7’s function [79], Fbw7β may have a prominent role in mitosis, and also most likely in cells arrested in response to various stresses. Further investigation is required to determine if this is the case or not.

Like Fbw7, PKC is often mutated in human cancers. It was recently demonstrated that the majority of mutations identified throughout the PKC family are loss of function, indicating a general role for PKCs as tumor suppressors [80]. The PKC-mediated stabilization of Fbw7α might therefore contribute to the tumor suppressive functions of PKC.

## Supporting information

S1 Fig Sequence alignment of the amino-terminal moieties of xFbw7 and hFbw7 isoforms. A. Sequences were obtained from UniProt, RefSeq or GenBank, aligned using the Clustal Omega tool of the UniProt server (http://www.uniprot.org/align/), and sequences were displayed with Jalview (http://www.jalview.org/). The sequences are as follows: Hs-FBW7 alpha (Q969H0), Hs-FBW7 beta (BAA91986.1), Hs-FBW7 gamma (Q969H0-4), Xl-FBW7 alpha (L8B5P6), Xl-FBW7 beta (XP_018105049.2), Xl-FBW7 gamma (XP_018105041.1). Primers for subcloning and PCR amplification of *X*. *laevis* FBW7 cDNA isoforms were designed according to the sequences available in databases with the following RefSeq accession numbers: XM_018249534.1 (alpha); XM_018249560 (beta); XM_018249552 (gamma). B. Expression of xFbw7 isoforms mRNA during *Xenopus* oocyte maturation. Semi-quantitative PCR analysis was performed on pCS2-xFbw7α, β or γ (Vc), total RNA of stage VI or mature (MII) oocytes. The level of the 18S mRNA remains fairly constant throughout early development and thus serves as a control.
(TIF)

S2 Fig Characterization of Fbw7 antibodies. A. Immunoprecipitation (IP) of xFbw7α, β and γ isoforms. xFbw7 isoforms were translated *in vitro* in the presence of [^35^S]-methionine and immunoprecipitated with specific anti-Fbw7 antibodies for phosphorimaging and for immunoblotting analysis with anti-Fbw7 antibodies. (i) and (s) designate the radiolabelled protein input and supernatant, respectively. B. MII-arrested eggs were extracted with XB buffer supplemented (+) or not (-) with phosphatase inhibitors (PI) and subsequently treated with an excess of lambda protein phosphatase (λP). The asterisk indicates a non specific immunoreactive band; ivt: xFbw7α translated *in vitro*.
(TIF)

S3 Fig The Fbw7α-S18E mutant interacts with Skp1, ubiquitin or Usp28 as well as the S18A mutant. A. Fbw7 (IPFb) or control (IPc) immunoprecipitates from HeLa cells transfected with FLAG-Fbw7α-S18A or –S18E. Complexes between Fbw7α and endogenous Skp1 were analyzed by immunoblotting. Input 10% (i), supernatant after IP (s). B. *In vitro* translated [^35^S]-FLAG-hFbw7α-18A or -wt were incubated in MII-egg extracts and mixed with either GST or GST-ubiquitin bound to magnetic beads. Input represents 25% of the total extract (i), total beads (B). Complexes were analyzed by phosphorimaging and immunoblotting. C. Usp28 or control immunoprecipitates from HeLa cells transfected with HA-Usp28 were mixed with *in vitro* translated [^35^S]-Fbw7α-18A or -18E as indicated. Input (i) represents 10%. Complexes were analyzed by phosphorimaging and immunoblotting.
(TIF)

S4 Fig Structural prediction analysis of the human Fbw7α-wt protein. A. The propensity for the Fbw7αN-terminal domain (residues 1 to 165) to be disordered was predicted using a selection of the latest disorder prediction methods, which includes: IUPRED [81]; Espritz [82]; DISEMBL [83]; DISOPRED3 [84] and IntFOLD-DR [85]. B. A model of full-length Fbw7α, including the extended disordered domain, was constructed using the IntFOLD server [85]. Molecular graphics rendering was performed using PyMOL (www.pymol.org), showing the disordered and the dimerization domains in red, the F-box domain in green and the WD40 domain in blue.
(TIF)

S1 NC3Rs ARRIVE guidelines checklist (DOCX)

S1 Uncropped images (TIF)

S2 Uncropped images (TIF)

S3 Uncropped images (TIF)

S4 Uncropped images (TIF)
