# Supplementary figures and images for: The stability of Fbw7α in M-phase requires its phosphorylation by PKC
Source: PLoS One. 2017 Aug 29;12(8):e0183500. doi: 10.1371/journal.pone.0183500 (PMC5574586; doi:10.1371/journal.pone.0183500)

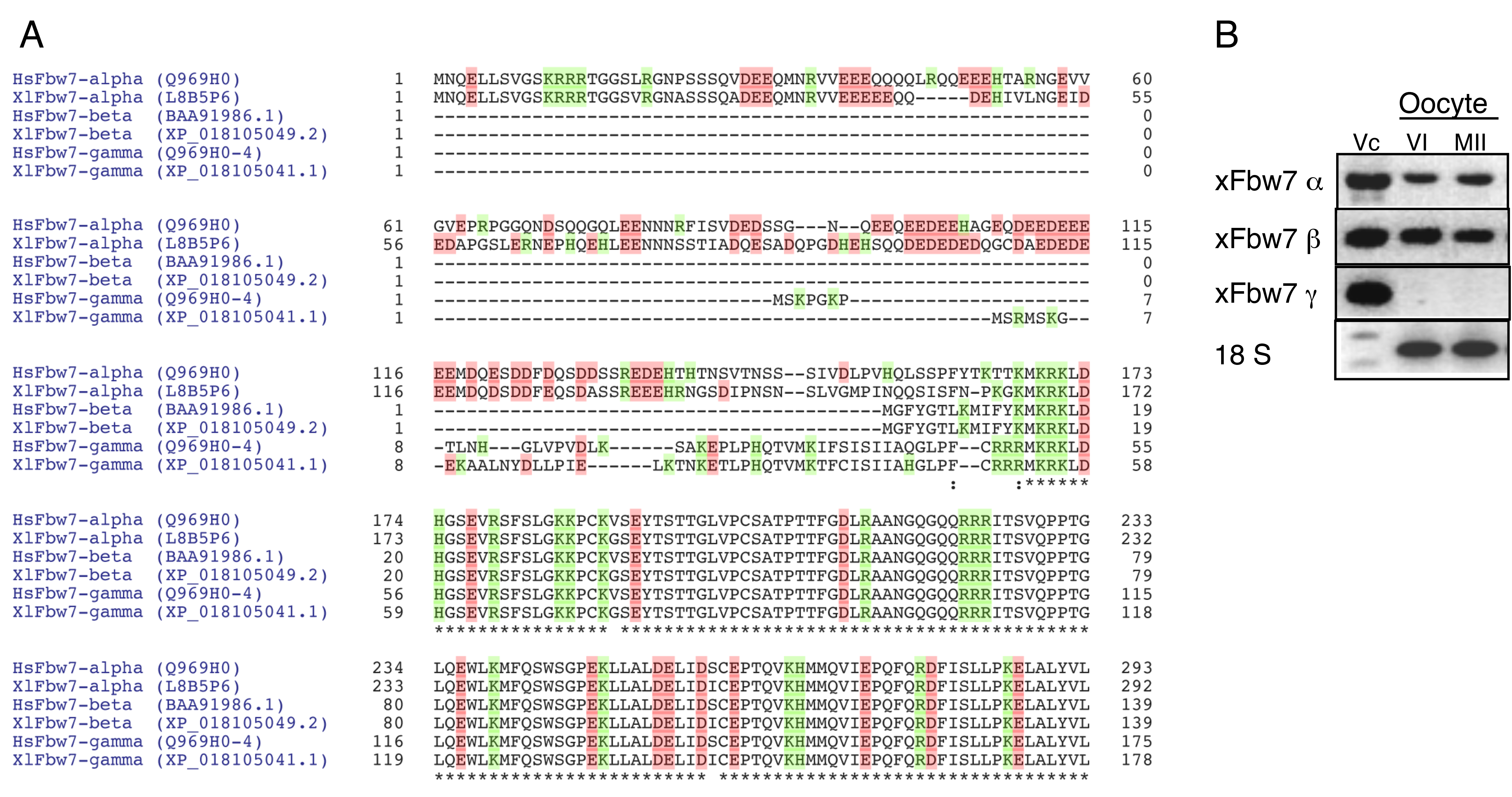

Supplement: S1 Fig — A. Sequences were obtained from UniProt, RefSeq or GenBank, aligned using the Clustal Omega tool of the UniProt server (http://www.uniprot.org/align/), and sequences were displayed with Jalview (http://www.jalview.org/). The sequences are as follows: Hs-FBW7 alpha (Q969H0), Hs-FBW7 beta (BAA91986.1), Hs-FBW7 gamma (Q969H0-4), Xl-FBW7 alpha (L8B5P6), Xl-FBW7 beta (XP_018105049.2), Xl-FBW7 gamma (XP_018105041.1). Primers for subcloning and PCR amplification of X. laevis FBW7 cDNA isoforms were designed according to the sequences available in databases with the following RefSeq accession numbers: XM_018249534.1 (alpha); XM_018249560 (beta); XM_018249552 (gamma). B. Expression of xFbw7 isoforms mRNA during Xenopus oocyte maturation. Semi-quantitative PCR analysis was performed on pCS2-xFbw7α, β or γ (Vc), total RNA of stage VI or mature (MII) oocytes. The level of the 18S mRNA remains fairly constant throughout early development and thus serves as a control. (TIF) [file pone.0183500.s001.tif]

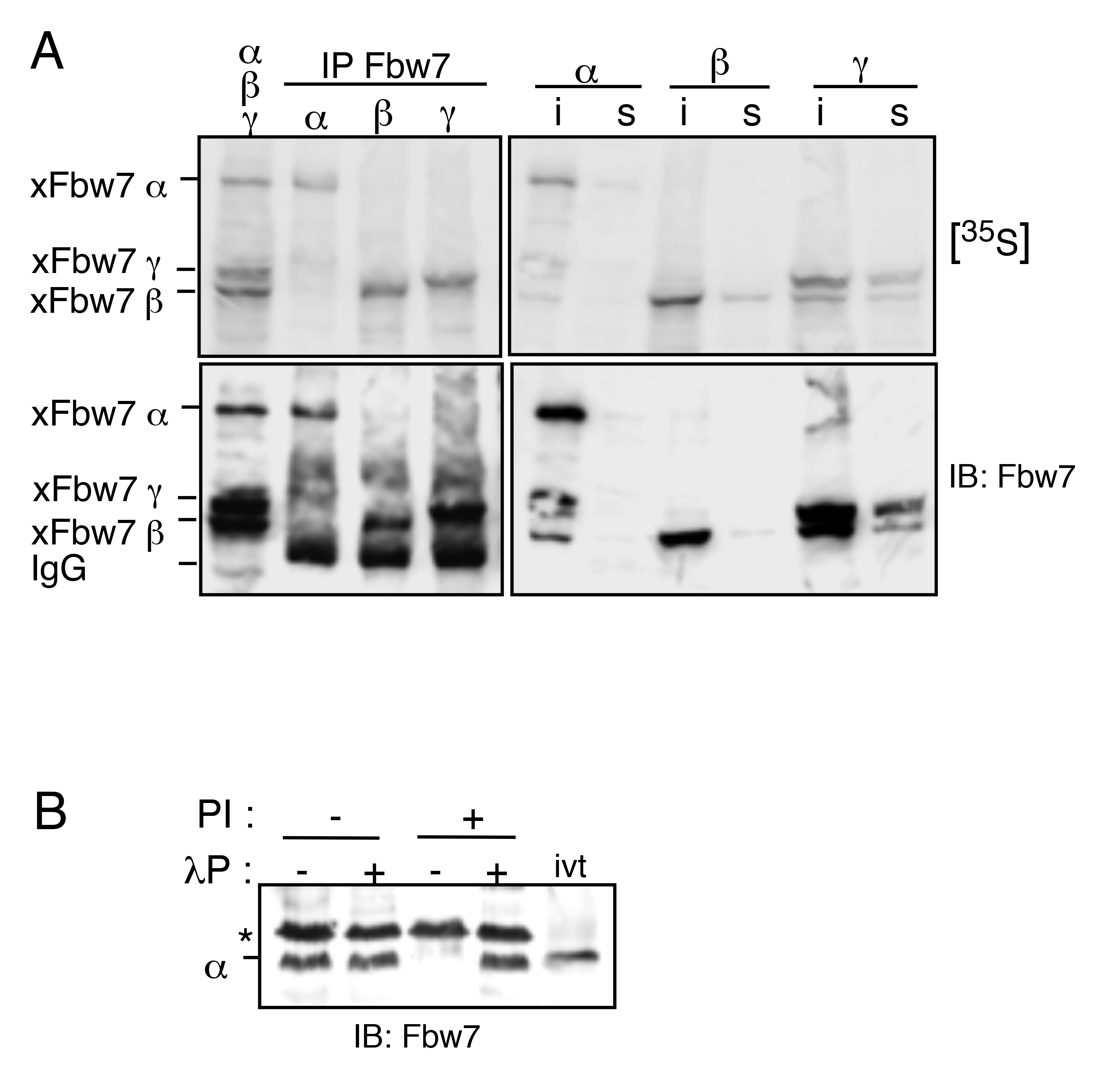

Supplement: S2 Fig — A. Immunoprecipitation (IP) of xFbw7α, β and γ isoforms. xFbw7 isoforms were translated in vitro in the presence of [35S]-methionine and immunoprecipitated with specific anti-Fbw7 antibodies for phosphorimaging and for immunoblotting analysis with anti-Fbw7 antibodies. (i) and (s) designate the radiolabelled protein input and supernatant, respectively. B. MII-arrested eggs were extracted with XB buffer supplemented (+) or not (-) with phosphatase inhibitors (PI) and subsequently treated with an excess of lambda protein phosphatase (λP). The asterisk indicates a non specific immunoreactive band; ivt: xFbw7α translated in vitro. (TIF) [file pone.0183500.s002.tif]

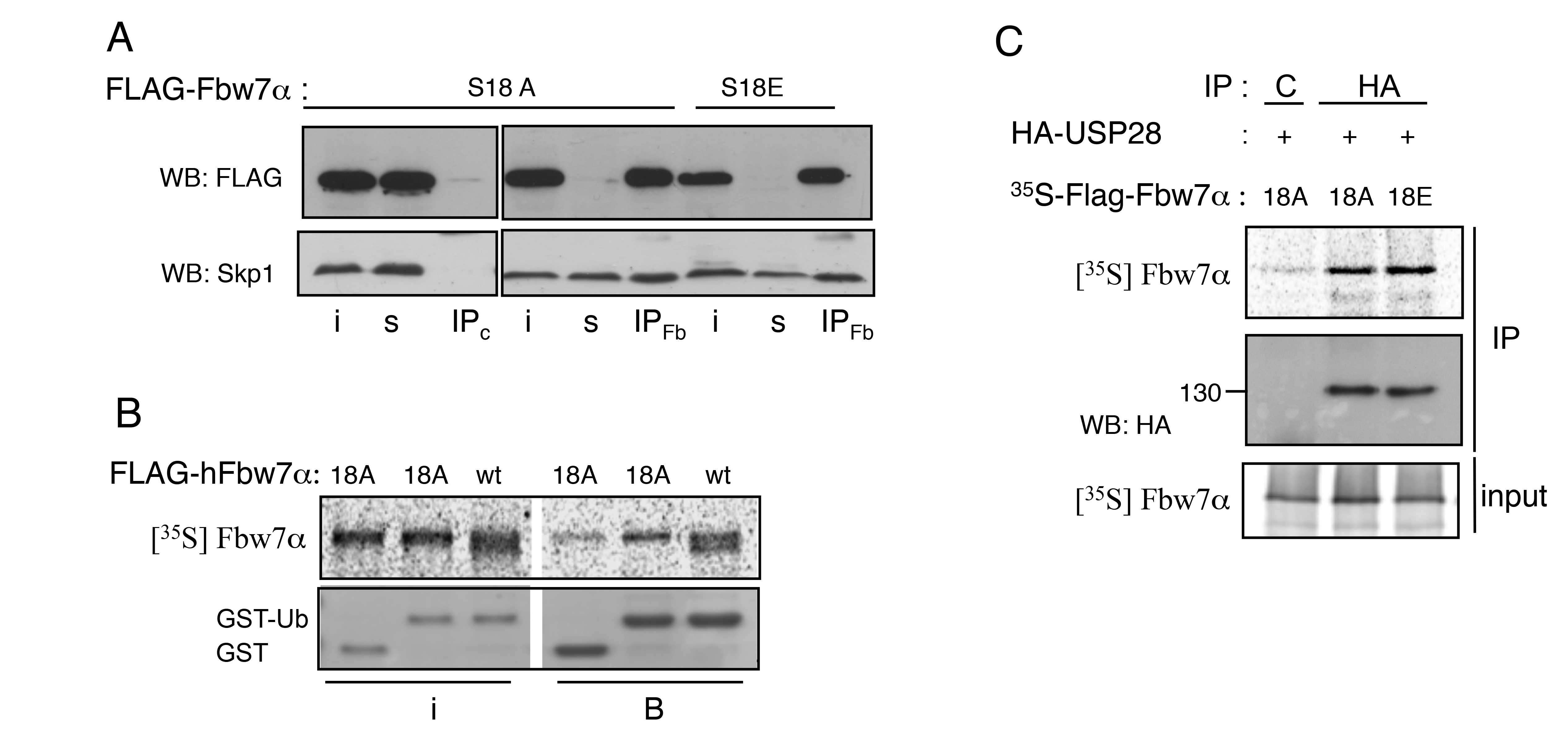

Supplement: S3 Fig — A. Fbw7 (IPFb) or control (IPc) immunoprecipitates from HeLa cells transfected with FLAG-Fbw7α-S18A or –S18E. Complexes between Fbw7α and endogenous Skp1 were analyzed by immunoblotting. Input 10% (i), supernatant after IP (s). B. In vitro translated [35S]-FLAG-hFbw7α-18A or -wt were incubated in MII-egg extracts and mixed with either GST or GST-ubiquitin bound to magnetic beads. Input represents 25% of the total extract (i), total beads (B). Complexes were analyzed by phosphorimaging and immunoblotting. C. Usp28 or control immunoprecipitates from HeLa cells transfected with HA-Usp28 were mixed with in vitro translated [35S]-Fbw7α-18A or -18E as indicated. Input (i) represents 10%. Complexes were analyzed by phosphorimaging and immunoblotting. (TIF) [file pone.0183500.s003.tif]

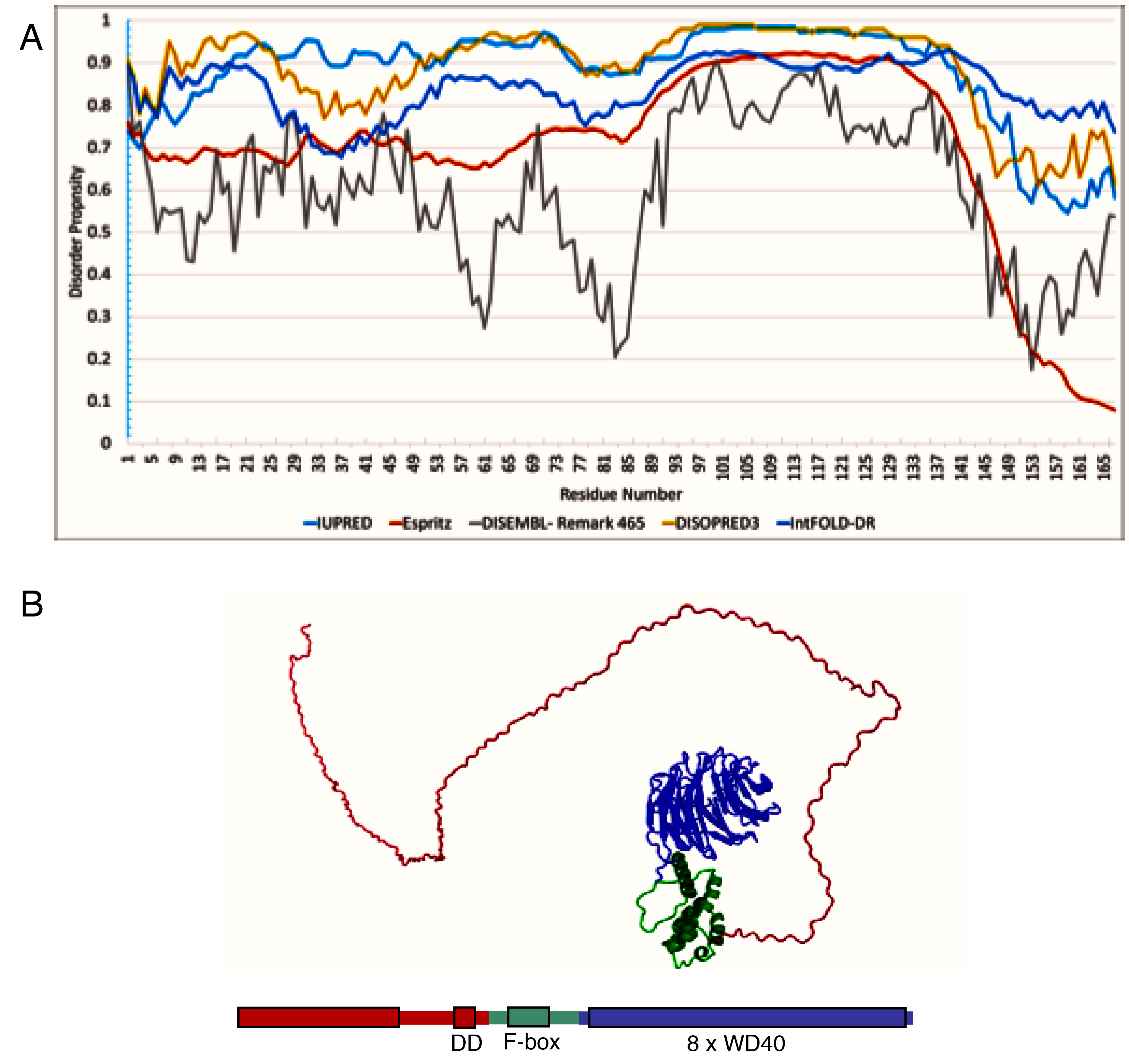

Supplement: S4 Fig — A. The propensity for the Fbw7αN-terminal domain (residues 1 to 165) to be disordered was predicted using a selection of the latest disorder prediction methods, which includes: IUPRED [81]; Espritz [82]; DISEMBL [83]; DISOPRED3 [84] and IntFOLD-DR [85]. B. A model of full-length Fbw7α, including the extended disordered domain, was constructed using the IntFOLD server [85]. Molecular graphics rendering was performed using PyMOL (www.pymol.org), showing the disordered and the dimerization domains in red, the F-box domain in green and the WD40 domain in blue. (TIF) [file pone.0183500.s004.tif]

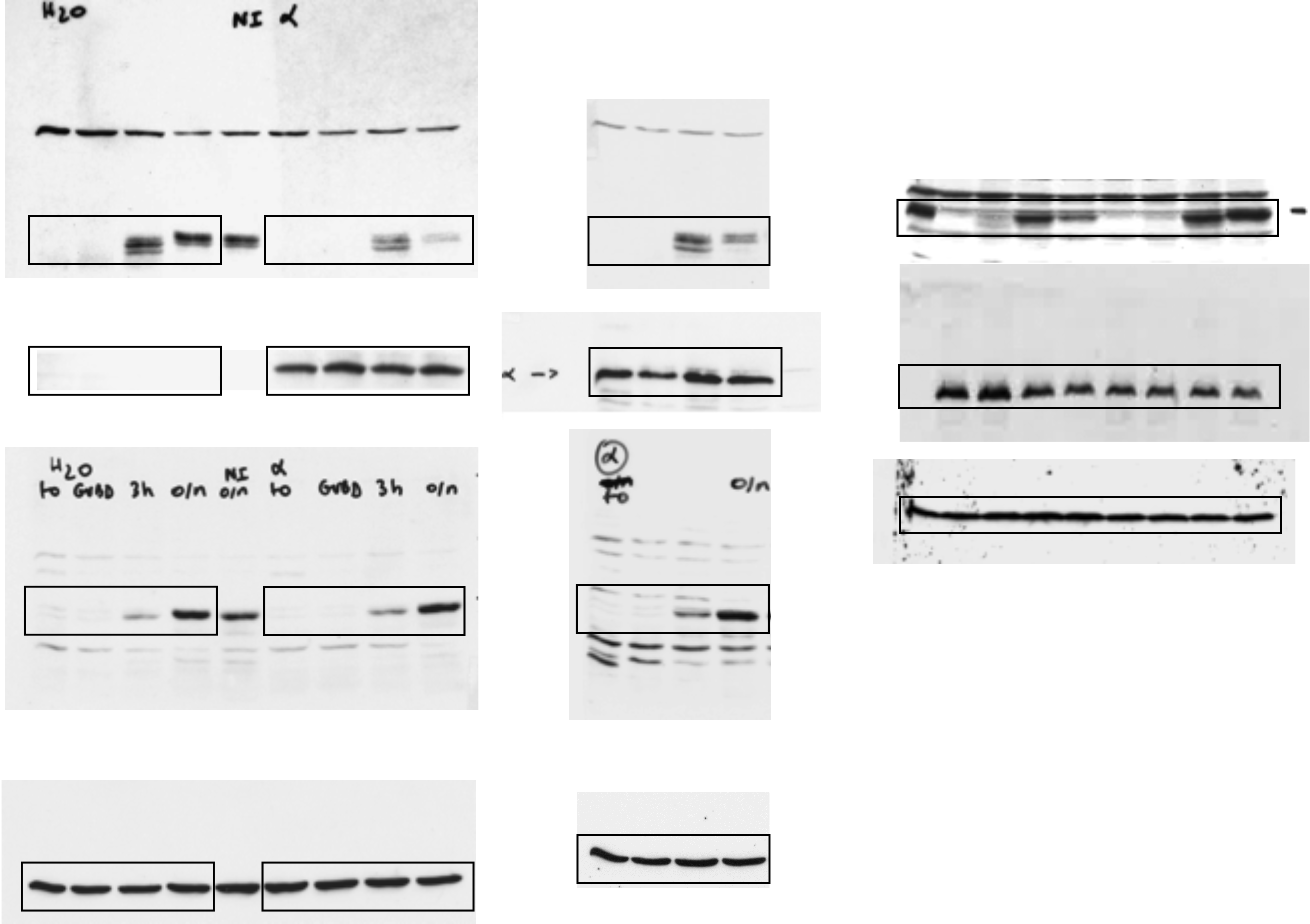

Supplement: S1 Uncropped images — (TIF) [file pone.0183500.s006.tif]

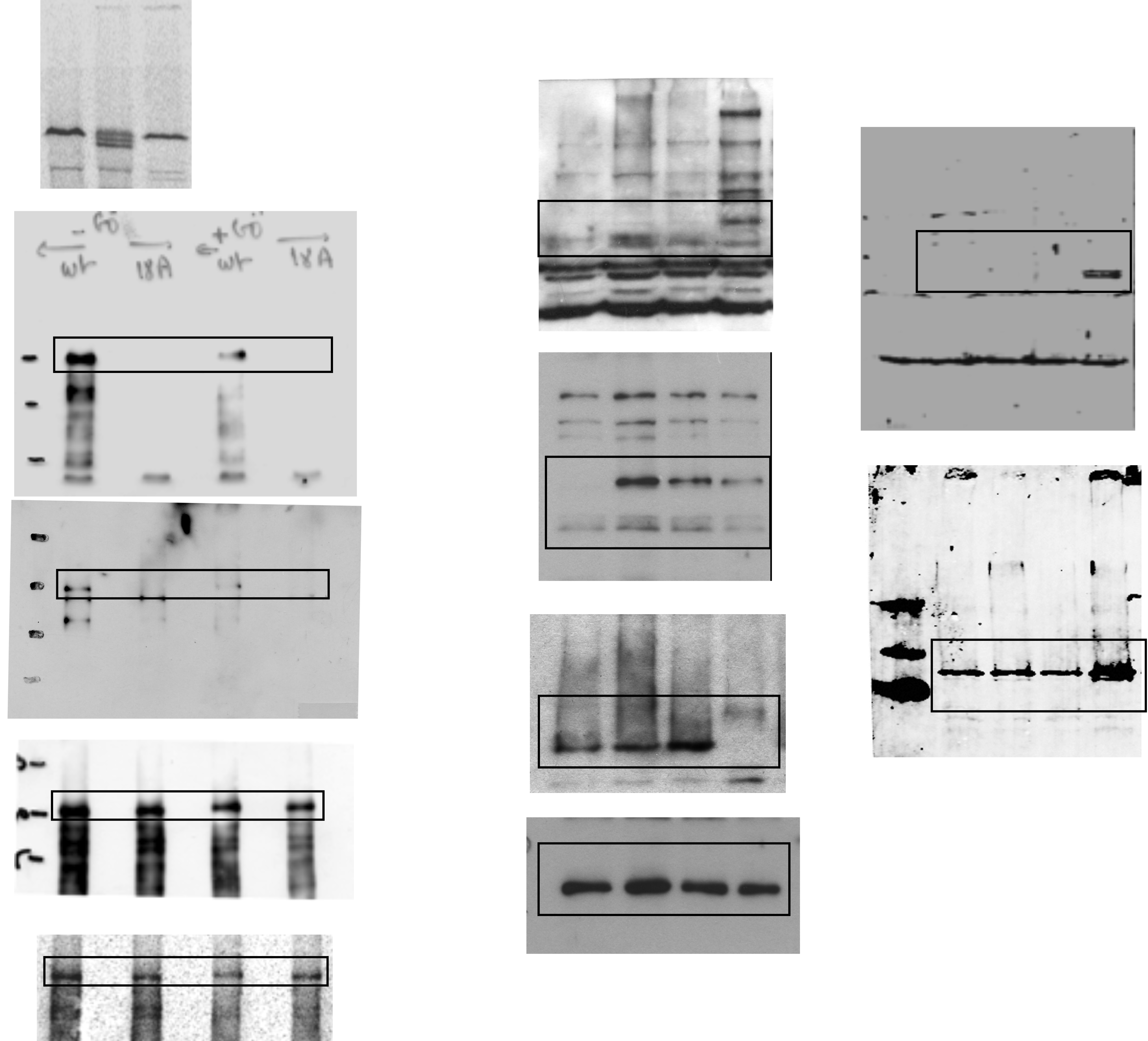

Supplement: S2 Uncropped images — (TIF) [file pone.0183500.s007.tif]

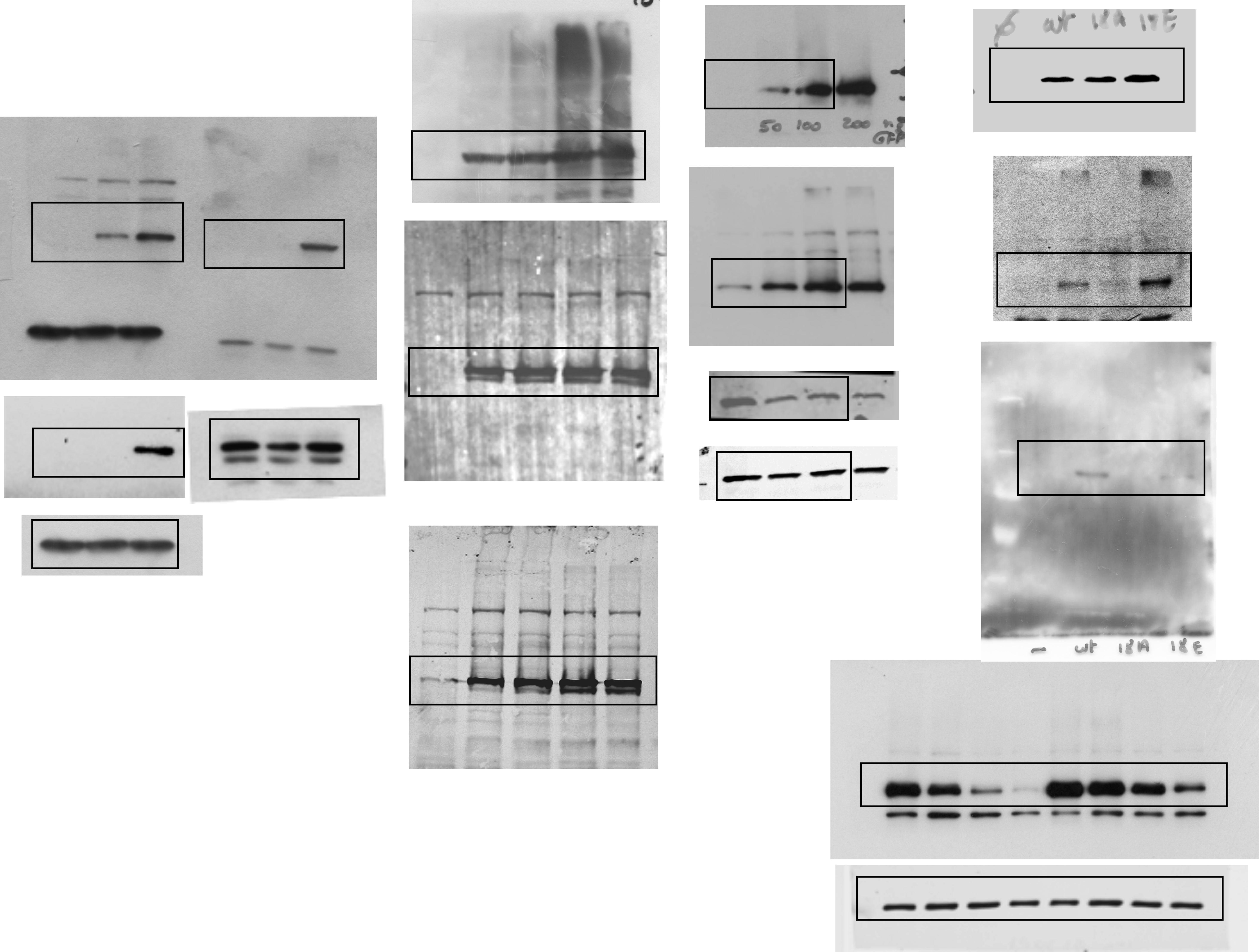

Supplement: S3 Uncropped images — (TIF) [file pone.0183500.s008.tif]

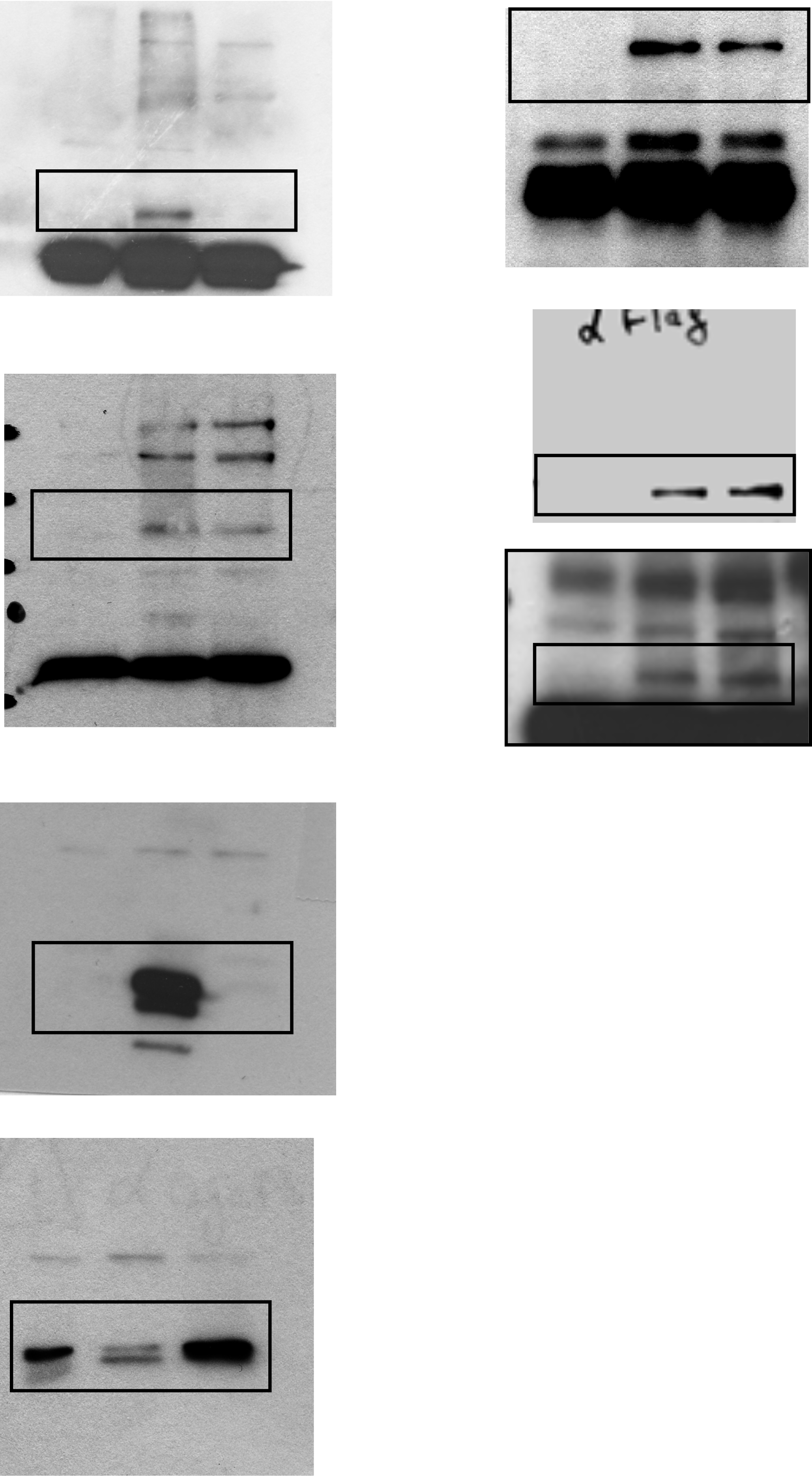

Supplement: S4 Uncropped images — (TIF) [file pone.0183500.s009.tif]
