# Supplementary material for: The stability of Fbw7α in M-phase requires its phosphorylation by PKC
Source: PLoS One. 2017 Aug 29;12(8):e0183500. doi: 10.1371/journal.pone.0183500 (PMC5574586; doi:10.1371/journal.pone.0183500)

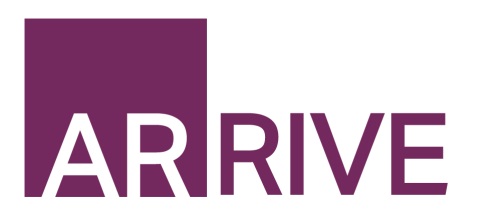


The ARRIVE Guidelines Checklist

Animal Research: Reporting In Vivo Experiments

Carol Kilkenny^1^, William J Browne^2^, Innes C Cuthill^3^, Michael Emerson^4^ and Douglas G Altman^5^

*^1^The National Centre for the Replacement, Refinement and Reduction of Animals in Research, London, UK, ^2^School of Veterinary Science, University of Bristol, Bristol, UK, ^3^School of Biological Sciences, University of Bristol, Bristol, UK, ^4^National Heart and Lung Institute, Imperial College London, UK, ^5^Centre for Statistics in Medicine, University of Oxford, Oxford, UK.*

|  | | ITEM | RECOMMENDATION | Section/ Paragraph |
| --- | --- | --- | --- | --- |
| **Title 1:** Fbw7, an F-box protein of the SCF ubiquitin ligase involved in the degradation of a myriad of key regulatory proteins, is phosphorylated by a PKC during M-phase, a phosphorylation event that leads to its inactivation. | | |  |  |
| **Abstract 2:** In this manuscript, we unravel a mechanism leading to the inactivation of the SCF Fbw7 E3-ligase. This regulation occurs specifically in *Xenopus* eggs arrested in meiosis allowing the accumulation of high levels of cyclin E, the first identified substrate of the SCF-Fbw7. We went on to show that this mechanism is conserved during the mammalian somatic cell cycle during mitosis to suppress SCF-Fbw7 E3-ligase activity and to stabilize Fbw7, this mechanism keeping Fbw7 in a resting inactive state. | | |  |  |
| INTRODUCTION | | |  |  |
| **Background 3:** We were interested in the regulation and functions of a key factor of the cell cycle machinery: cyclin E. Deregulated cyclin E expression has been linked to replicative stress and genomic instability. Its expression is periodic: it accumulates in late G1, peaks at G1/S, and declines during S phase. The ubiquitin ligase SCF associated with Fbw7 is crucial for the maintenance of cyclin E periodicity in normal cell cycles, since its functional inactivation leads to increased cyclin E levels at all cell cycle phases, including mitosis. However, accumulation of cyclin E during a mitotic-like phase does occur naturally during meiotic maturation of the *Xenopus* *laevis* oocyte, the final stage of oogenesis. We thus wanted to investigate the causes of this egg specificity.    We used *Xenopus* eggs and human cells in culture to know if this regulatory mechanism is conserved during somatic cell cycle. For all the experiments to be carried out, we used commercial antibodies as well as two antibodies that we produced at the beginning of the study because they were not commercialized. It was essential to be able to obtain such tools to enable us to progress in our investigation. | | |  |  |
| **Objectives 4:** The primary objective of the study was to determine whether Fbw7 is expressed or not in eggs. Since the answer was yes, we explored the mechanism leading to its inactivation and the accumulation of its target cyclin E in mature oocytes. | | |  |  |
| METHODS | | |  |  |
| **Ethical statement 5:** Two antibodies were produced at the beginning of this study from rabbits dedicated to the production of antibodies. These rabbits were handled in the animal house of the Institut Universitaire de Technologie de Montpellier, which has an institutional agreement (number D34-172-8) from Direction Départementale de la Protection des Populations (DDPP) de l’Hérault (France), operating under the supervision of the Ministry of Agriculture and dedicated to rabbit immunization and blood sampling.  *Xenopus laevis* frogs were housed at the CRBM (Centre de Recherche de Biologie Cellulaire de Montpellier), which has an institutional agreement (number A34-172-39). The protocols were not further submitted to the approval of an ethics committee, as such approval was not necessary for those experiments under the French and European legislation at the time they were conducted. | | |  |  |
| **Study design 6:** For each antibody, a single rabbit was immunized according to an experimental protocol of 6 months.  For our experiments using *Xenopus leavis* oocytes, we used 2 to 3 females per experiment. | | |  |  |
| **Experimental procedures 7:**  Rabbit immunization protocol  The experimental protocol begins with a subcutaneous injection at: Day 1: This 0.8 ml injection contains 0.4 ml complete Freund’s adjuvant + 0.4 ml aqueous solution with antigens (100 μg of antigens). Day 7: 2th injection: 0.4 ml incomplete Freund’s adjuvant + 0.4 ml aqueous solution with antigens (100 μg antigen). Day 28: 3th injection: 0.4 ml incomplete Freund’s adjuvant + 0.4 ml aqueous solution with antigens (100 μg antigen). Day 35: 1st blood sample of 10 ml of blood to the ear of the rabbit. Same as day 42 and 49 for the 2nd and 3rd samples. Day 56: 4th injection: 0.4 ml incomplete Freund’s adjuvant + 0.4 ml aqueous solution with antigens (100 μg antigen). Days 63, 70 and 77: 4th, 5th, and 6th specimens (10 ml of blood to the ear). Day 84: 5th immunization: 0.4 ml incomplete Freund’s adjuvant + 0.4 ml aqueous solution with antigen (100 μg of antigens). Days 91, 98, 105: 7th, 8th and 9th specimens (10 ml of blood to the ear). Days 112: 6th immunization: 0.4 ml incomplete Freund’s adjuvant + 0.4 ml aqueous solution with antigens (100 μg antigen). Days 119,126 and 133: 10th, 11th and 12th specimens (10 ml of blood to the ear). Day 140: 7th and last immunization Day 147: 13th withdrawal Day 154: Euthanasia. During this stage we can take between 120 and 150ml of blood.  At the end of the immunization protocol, rabbits were anesthetized with Pentobarbital (30 mg/kg) and then sacrificed by intra-cardiac injection of Dolethal (Vetoquinol S.A.) 1.1 ml/kg.  *Xenopus laevis frogs*  Mature oocytes were collected from females that had been injected with 0.5 ml of human chorionic gonadotropin (hCG) - 500 IU into the dorsal lymph sac. Females begin laying eggs 12 hours after the hCG injection. Frogs are used again after a recovery period of 6 months. After this period, the animals can be used again for a new egg laying. This cycle may last for several years until the female shows a lack of appetite or weight loss. In this case, *Xenopus laevis* frogs were euthanized by immersion in a buffered solution of Tricaine (MS222 4 g/l).  The procedure to remove oocytes of stages VI was conducted under Tricaine anesthesia (MS222 2g/l) to minimize animal suffering. The ovarian lobes were removed through a small abdominal incision. Frogs were then left to recover in a separate tank and were frequently monitored until conscious and were returned to their original tanks the following day. Surgical oocyte harvest was performed once a year on each frog for up to three years. After this period, *Xenopus laevis* frogs were euthanized by immersion in a buffered solution of Tricaine (MS222 4 g/l). | | |  |  |
| **Experimental animals 8:**  Rabbits  The rabbits used were adults of about 4 months. When the rabbit arrives at the sampling stage (after 15 days of adaptation and one month in order to carry out the 3 injections), its weight is mostly greater than 2.5 kg.  Rabbits (New Zealand albinos) were obtained from CEGAV S.S.C, Les Passeries, 61350 Saint Mars d’Egrenne, France.  *Xenopus laevis frogs*  Females were purchased from the Centre d’Elevage de Xenopes, Campus de Beaulieu, UMS 3387, av. du Général Leclerc, 35042 Rennes, France. | | |  |  |

The ARRIVE guidelines. Originally published in *PLoS Biology*, June 2010^1^

| **Housing and husbandry 9:**  Rabbits  Our rabbits were housed in individual cages. An independent room was dedicated to their accommodation.  *Xenopus laevis* frogs  The frogs were housed in glass tanks (Techniplast) filled with filtered water, which was monitored daily for pH, and maintined at a temperature of 19°C. The photoperiod was set to 12 hr ON, 12 hr OFF. Frogs were fed twice per week with Aquatic 3 granules (Mazuri). Two separate rooms were dedicated for rest and experimentation. |  | |
| --- | --- | --- |
| **Sample size 10:**  Rabbits  The experimental protocol used allows us both to obtain the quantity of antibody sufficient to use only one rabbit per antigen and to know when the efficiency of the antibody was optimum.  *Xenopus laevis* frogs  We use 2 to 3 females per experiment depending on the number of eggs we wish to obtain. To make sure we have a laying we inject two to three females, as egg laying is never equivalent in terms of quality / quantity of oocytes. |  | |
| 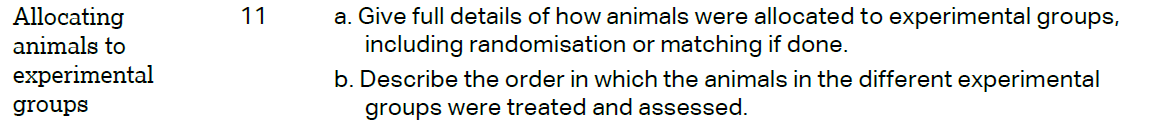 | N/A | |
| 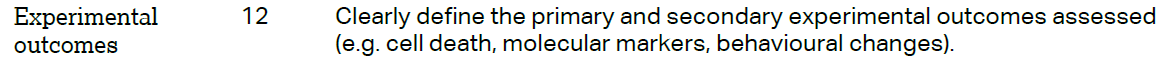 | N/A | |
| 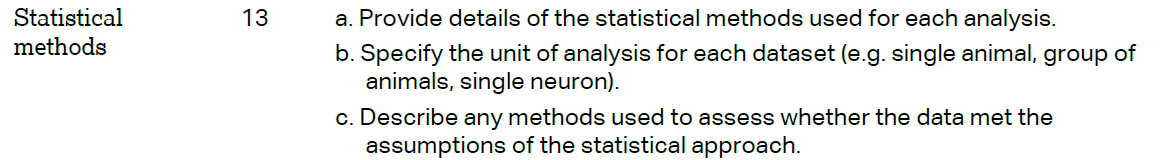 | N/A | |
| RESULTS |  | |
| **Baseline data 14 :** We obtain two specific antibodies that allowed us to demonstrate that the F-Box Fbw7 is expressed in eggs and phosphorylated at a specific residue by a kinase PKC. Furthermore, these antibodies were able to recognize the human Fbw7 protein. |  | |
| 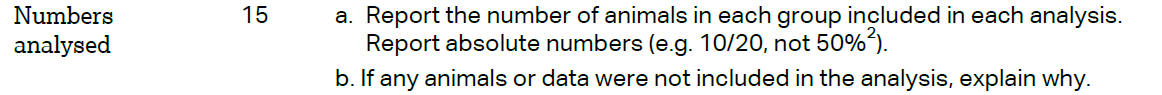 | N/A | |
| 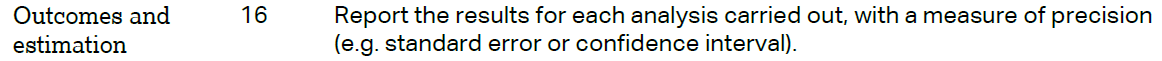 | N/A | |
| 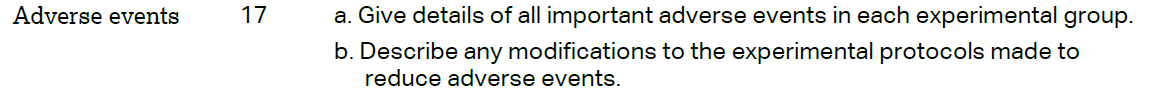 | N/A | |
| DISCUSSION |  | |
| **Interpretation/ Scientific implications 18:**  The results of this study allowed us to unravel a mechanism implicating PKC in an evolutionarily-conserved pathway that aims to protect Fbw7α from degradation by keeping it transiently in a resting, inactive state. These findings provide the demonstration of a physiologic regulation of Fbw7α, which is a well recognized and important human tumor suppressor. |  | |
| **Generalisability/translation 19:**  The use of *Xenopus* eggs is justified by the quantity of oocytes laid (2000), a perfect synchronization at a precise stage of cell division (all these oocytes are naturally arrested in metaphase II of meiosis). Furthermore, working on *Xenopus* oocytes requires a simple subcutaneous injection of the frogs twice a year.  The results obtained with the *Xenopus* eggs have provided a strong basis for the study in human cells in which we next demonstrated that Fbw7 regulation is conserved.  Relevance to human biology: We show in this study that PKC is a limiting factor contributing to Fbw7α stability. It is at this level that the information of our manuscript is important since it was recently shown that PKC mutations in cancer are loss of function: the destabilization of Fbw7α could be one of the mechanisms explaining why inhibiting PKC has been detrimental in cancer clinical trials. Our data underscore the need for therapies that restore PKC activity in cancer cells. |  | |
| **Funding 20:**  The project was supported by the CNRS. The authors received no other specific funding for this work. | |  |


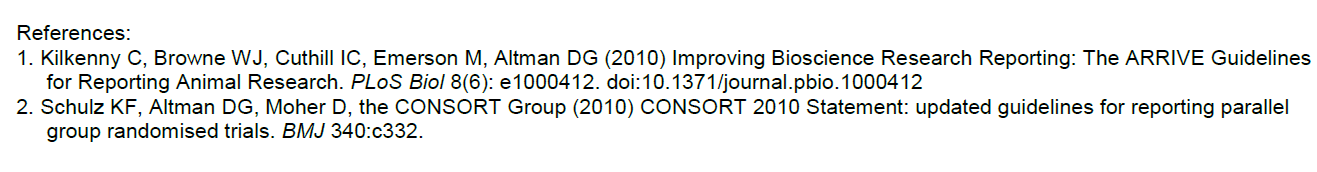

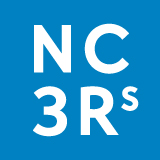

Supplement: S1 NC3Rs ARRIVE guidelines checklist — (DOCX) [file pone.0183500.s005.docx]
